# Lipid response patterns in acute phase paediatric *Plasmodium falciparum* malaria

**DOI:** 10.1007/s11306-017-1174-2

**Published:** 2017-02-23

**Authors:** Judy Orikiiriza, Izabella Surowiec, Elisabeth Lindquist, Mari Bonde, Jimmy Magambo, Charles Muhinda, Sven Bergström, Johan Trygg, Johan Normark

**Affiliations:** 1 Infectious Diseases Institute, College of Health Sciences, Makerere University, P.O. Box 22418, Kampala, Uganda; 2 Department of Immunology, Institute of Molecular Medicine, Trinity College Dublin, St. James’s Hospital, Dublin, 8 Ireland; 3 Rwanda Military Hospital, P.O. Box: 3377, Kigali, Rwanda; 4 Computational Life Science Cluster (CLiC), Department of Chemistry, Umeå University, 901 87 Umeå, Sweden; 5 Department of Molecular Biology, Umeå University, 901 87 Umeå, Sweden; 6 Department of Chemistry, Umeå University, 901 87 Umeå, Sweden; 7 Department of Immmunology and Microbiology, School of Biomedical Sciences College of Health Sciences, Makerere University, P.O Box 7072, Kampala, Uganda; 8 Laboratory for Molecular Infection Medicine Sweden (MIMS), 901 87 Umeå, Sweden; 9 Umeå Center for Microbial Research, 901 87 Umeå, Sweden; 10 Division of Infectious Diseases, Department Clinical Microbiology, Umeå University, 901 87 Umeå, Sweden

**Keywords:** Lipidomics profiling, Malaria, *Plasmodium falciparum*, Triacylglycerides, Lysophosphatidylcholines

## Abstract

**Introduction:**

Several studies have observed serum lipid changes during malaria infection in humans. All of them were focused at analysis of lipoproteins, not specific lipid molecules. The aim of our study was to identify novel patterns of lipid species in malaria infected patients using lipidomics profiling, to enhance diagnosis of malaria and to evaluate biochemical pathways activated during parasite infection.

**Methods:**

Using a multivariate characterization approach, 60 samples were representatively selected, 20 from each category (mild, severe and controls) of the 690 study participants between age of 0.5–6 years. Lipids from patient’s plasma were extracted with chloroform/methanol mixture and subjected to lipid profiling with application of the LCMS-QTOF method.

**Results:**

We observed a structured plasma lipid response among the malaria-infected patients as compared to healthy controls, demonstrated by higher levels of a majority of plasma lipids with the exception of even-chain length lysophosphatidylcholines and triglycerides with lower mass and higher saturation of the fatty acid chains. An inverse lipid profile relationship was observed when plasma lipids were correlated to parasitaemia.

**Conclusions:**

This study demonstrates how mapping the full physiological lipid response in plasma from malaria-infected individuals can be used to understand biochemical processes during infection. It also gives insights to how the levels of these molecules relate to acute immune responses.

**Electronic supplementary material:**

The online version of this article (doi:10.1007/s11306-017-1174-2) contains supplementary material, which is available to authorized users.

## Introduction

*Plasmodium falciparum* malaria remains a major global health and economic burden in spite of recent intense preventive measures. Malarial disease entails a complex range of parasite-host interactions and a dynamic flow of immune responses affecting both organisms. The parasite relies on an exchange of metabolites with the human host to ensure survival and proliferation (Olszewski et al. 2009; Kafsack and Llinas 2010; Lakshmanan et al. 2011). The host acute phase immune response to the parasite carries many common denominators with other acute infectious and inflammatory conditions such as sepsis and systemic inflammatory response syndrome (SIRS) (O’Donnell et al. 2009).

Lipids and lipoprotein metabolism in humans have been the focus of intense study and are extensively described in many books and reviews (Vance and Vance 2008; Brown and Marnett 2011). Recently, emphasis has been put on the role of lipoproteins in relation to the immune system, and the acute phase response in particular. Feingold et al. established that the administration of lipopolysaccharides (LPS) increases lipoprotein levels in the peripheral circulation (Feingold et al. 1992). Hyperlipidemia has been described to accompany different infectious and inflammatory diseases (Gallin et al. 1969; Alvarez and Ramos 1986; Cabana et al. 1989; Khovidhunkit et al. 2004; Wendel et al. 2007). It is also known that hyperlipidaemia in infection arises because of an increase of very low density lipoproteins (VLDL) levels due to increased de novo fatty acid (FA) synthesis and suppressed fatty acid oxidation. This results in increased hepatic production of VLDL, suppression of VLDL lipolysis by inhibition of lipoprotein lipase and increased adipose tissue lipolysis. The proposed mechanisms by which hyperlipidaemia affects the immune response to infection involves dissolution of toxins combined with their neutralization and also the immunomodulatory role of lipoproteins (Barcia and Harris 2005; Navab et al. 2005). One of the integral components of VLDL, cholesterol, is necessary for the internalization of eukaryotic pathogens into host cells (Bansal et al. 2005).

Several studies have observed serum lipid changes during malaria infection in humans. A meta-analysis study in 2013 (Visser et al. 2013) concluded that cholesterol, high density lipoproteins (HDL) and low density lipoproteins (LDL) concentrations are lower in malaria compared to both healthy controls and to other febrile diseases. TAGs were shown to be elevated during malaria infection compared to healthy controls, but not statistically significant compared to symptomatic controls. A recent study reported however that lipoprotein levels were specifically perturbed by malaria infection as compared to healthy controls, encephalitis and sepsis (Sengupta et al. 2016). The clearance of low-level *P. falciparum* infection has also been shown to normalize these changes (Faucher et al. 2002). Although the quantity of lipid changes seems to be related to the severity of malaria in some studies (Parola et al. 2004; Sengupta et al. 2016), others found no correlation (Baptista et al. 1996; Kittl et al. 1992). Availability of lipoproteins in malaria infection was reported to be important for adherence of infected erythrocytes to the microvasculature (Frankland et al. 2006, 2007). In a recent study we have found increased levels of fatty acids in malaria patients compared with controls, which could also differentiate between mild and severe cases and were positively correlated with parasitaemia values (Surowiec et al. 2015). Perturbations in the lipid metabolism have also emerged as a defining factor to survival in sepsis (Langley et al. 2013). A profound analysis of lipid metabolism during malaria infection holds promise for deeper understanding of the disease.

Although literature regarding lipid changes connected to malaria infection is vast, it has been mainly focused on measurement of lipoprotein fractions. There is an extensive literature describing lipid analysis using mass spectrometry in malaria in vitro studies (Vo Duy et al. 2012; Botté et al. 2013; Gulati et al. 2015; Shears et al. 2017), parasites themselves (Maréchal et al. 2011) and in mice models (Itoe et al. 2014), but so far no study have described the application of lipidomics in malaria to clinical samples. Lipidomics can be described as a global lipid analysis and focuses on the identification and relative (untargeted lipidomics) or absolute (targeted lipidomics) quantification of all lipid species present in biological samples, followed by characterization of samples in relation to the scientific hypothesis. Identification of chemical species that could be specifically connected to infection can be important not only from the point of understanding the pathogenesis and impact on the immune response, but also suggest possible avenues of treatment.

The aim of the present study was to identify novel patterns of lipids in malaria patients using a high resolution approach based on lipid-targeted plasma extraction and followed by Liquid Chromatography Mass Spectroscopy-Quadrupole Time of Flight (LCMS-QTOF) profiling and identification of lipid species. At the same time, we wanted to map the range of lipid species in relation to available clinical and personal parameters of the patients. We also assessed relation of levels of lipid species to parasitaemia values in order to better understand the host response in correlation to incremental parasite burden. The expectation was to enhance our knowledge about biochemical processes involving lipid species that take place during malaria infection.

## Materials and methods

### Patients

The research was carried out according to The Code of Ethics of the World Medical Association (Declaration of Helsinki). Ethical clearance was obtained from the Rwanda National Ethics Committee RNEC (No: 279/RNEC/2010) and the Regional Ethical Committee in Umeå (No: 09-064). Written informed consent was provided by the parent or legal guardian of each participant. The patients were assessed on site by the attending paediatrician/study medical officer and biometric and clinical parameters were recorded, as listed in Tables S1–S3 and summerized in Table 1. The patients were categorized according to the WHO categories of severe malaria as well as mild malaria (WHO 2010). Patients that were treated for malaria, had a known HIV positive status or jaundice were excluded from the study. This was done to avoid confounding of the metabolic profiles that could be related to treatment and both mentioned diseases with the malaria profile. Treatment, HIV and jaundice are expected to influence the metabolic plasma profile, which in the limited sample set studied would not be possible to resolve from malaria one. The patients had been fed ad libitum before sampling. Blood samples were drawn on site and assessment of parasite presence was done in the routine lab facilities coupled to each clinic in Rwanda and in Sweden of saved Giemsa stained thin blood smears. Twenty samples from each group of diagnostic categories were selected (healthy controls, mild and severe malaria), ten from each gender out of 690 patients included in the cohort, using a full factorial design as described in (Surowiec et al. 2015) and in Supplementary Material. The main aim of the applied sample selection procedure was to select the samples that would uniformly span the space described by all available samples and personal and clinical parameters recorded for them.

**Table 1:** Selected clinical and personal parameters of malaria patients included in the study

Clinical parameter	All infected (39 individuals)	Mild cases (19 individuals)	Severe cases (20 individuals)
Parasitaemia	M: 1.9, IQR: 5.4, TR: 0.0-26.6	M: 1.3, IQR: 0.9, TR: 0.2-4.0	M: 6.0, IQR: 9.0, TR: 0-26.6
Age (months)	M: 51, IQR: 21, TR: 18–72	M: 48, IQR: 21, TR: 23–72	M: 54, IQR: 25, TR: 18–72
HC (cm)	M: 50, IQR: 2, TR: 42–54	M: 50, IQR: 3, TR: 42–54	M: 50, IQR: 4, TR: 45–52
Weight	M: 15, IQR: 6, TR: 10–22	M: 15, IQR: 4, TR: 10–22	M: 14.5, IQR: 5, TR: 11–22
Height	M: 100, IQR: 13, TR: 44–130	M: 99, IQR: 14, TR: 80–130	M: 101, IQR: 14, TR: 44–115
MUAC (cm)	M: 16, IQR: 2.5, TR: 13.5–18	M: 16, IQR: 3, TR: 13.5–18	M: 16, IQR: 2.0, TR: 13.5–17.5
Temperature	M: 38.4, IQR: 2, TR: 35.7–40.1	M: 38.3, IQR: 2, TR: 36.3–40.0	M: 38.4, IQR: 2, TR: 35.7–40.1
Systolic pressure	M: 100, IQR: 15, TR: 85–120	M: 100, IQR: 20, TR: 85–120	M: 103, IQR: 11, TR: 90–120
Diastolic pressure	M: 70, IQR: 20, TR: 50–98	M: 60, IQR: 19, TR: 50–80	M: 75, IQR: 27, TR: 50–98
Pulse rate	M: 118, IQR: 44, TR: 72–192	M: 100, IQR: 22, TR: 72–170	M: 139, IQR: 46, TR: 96–192
Breathing rate	M: 28, IQR: 12, TR: 20–64	M: 25, IQR: 7, TR: 20–60	M: 30, IQR: 17, TR: 24–64
Haemoglobin	M: 10.7, IQR: 2.3, TR: 6.3–13.9	M: 11.1, IQR: 2.7, TR: 7.2–13.2	M: 10.6, IQR: 2.1, TR: 6.3–13.9
Glucose	M: 6.2, IQR: 2.5, TR: 1.7–8.1	M: 5.5, IQR: 2.0, TR: 3.6–8.1	M: 6.4, IQR: 1.8, TR: 1.7–7.4
Length of illness (days)	M: 2, IQR: 2, TR: 1–6	M: 2, IQR: 2, TR: 1–4	M: 2, IQR: 2, TR: 1–6
Gender	19 females, 20 males	9 females, 10 males	10 females 10 males
Cough	19 positive	9 positive	10 positive
Diarrhea	8 positive	3 positive	5 positive
Breathlessness	6 positive	0 positive	6 positive
Loss of consciousness	3 positive	0 positive	3 positive
Black urine	2 positive	0 positive	2 positive
Convulsions	4 positive	0 positive	4 positive
Illness apart malaria	14 positive	6 positive	8 positive
Prostration	15 positive	0 positive	15 positive
Splenomegaly	4 positive	0 positive	4 positive
Hepatomegaly	7 positive	2 positive	5 positive
Dehydration/dry mucus	7 positive	1 positive	6 positive
Depth of breathing	1: 1 ind.; 2: 12 ind.; 3: 24 ind	1: 1 ind.; 3: 17 ind	2: 12 ind.; 3: 7 ind

*TR* total range; *M* median; *Ind*. individual; *HC* head circumference; *MUAC* mid-upper arm circumference

### Lipidomics analysis

Plasma was prepared through gel flotation of whole blood on site in Rwanda, snap frozen and transported in liquid nitrogen to Sweden, thawed once for aliquoting, frozen and stored in −80 °C until plasma lipids were extracted with modified Folch extraction and analysed with LCMS-QTOF as described in Supplementary Material. Six pooled plasma samples were analysed in parallell with the samples from patients in order to establish repeatability of the analytical procedure. The average relative standard deviation (RSD) for all the metabolites detected in the samples was 5.2%. This result combined with low average RSDs of nine internal standards from all samples (5.3%) proves the stability of the applied analytical procedure. Example total ion chromatograms for each class of samples are presented at Fig. S1.

### Compound identification

A targeted feature extraction of the acquired LCMS-QTOF data was performed using the Profinder™ software package, version B.06.00 and in-house retention time and mass spectra library built from standards of pure compounds and from compounds that were identified with MS/MS based on the known lipid fragmentation patterns in the commercial lipid mixtures from Sigma–Aldrich (Stockholm, Sweden). After peak extraction, each compound was manually checked for accurate mass and retention time agreement with appropriate standards from the library; peaks with bad characteristics (overloaded, noisy, not Gaussian etc.) were excluded from the analysis. Identification of the compounds was confirmed by comparison of their MS/MS spectra with MS/MS spectra of relevant compounds from the library. We managed to identify 117 lipid molecules (Table S4). Examples MS/MS spectra for the compounds from main detected lipid classes (LPCs, PCs, TAGs and SMs) are presented at Fig. S2.

### Data normalization

The peak areas representing the different lipid species were normalized using the areas from nine internal standards. These peaks were eluted during the entire chromatograph according to the following procedure. A principal component analysis model with Unit Variance Scaling (without subtraction of the average, UVN) was calculated using the peak areas of the internal standards. The t1-score value from this model for each sample was used to normalize the resolved data by dividing the peak areas of each sample with the corresponding score value (Redestig et al. 2009).

### Data processing and multivariate and univariate data analysis

Compound data was imported into the SIMCA software (version 14.0) from MKS Data Analytics Solutions (Umeå, Sweden) for multivariate analysis. The data was mean centred and scaled to unit variance. Principal component analysis (PCA) was used to obtain an overview of the variation in the data and to check for trends and outliers in the data and orthogonal partial least squares discriminant analysis (OPLS-DA^®^) was used to compare lipid profiles of different classes of samples. Seven-fold cross-validation was used for calculating the all models. Lipidomic profiles related to parasitaemia were obtained from p(corr) vectors in the OPLS models with parasitaemia values as Y and lipid levels as X. In all cases maximally one orthogonal component was allowed in the OPLS models to avoid the risk of over-fitting (Trygg and Wold 2002). The significance of a metabolite for classification in the OPLS-DA models was specified by calculating the 95% confidence interval for the loadings using jack-knifing (Efron and Gong 1983).

One-way ANOVA and two-tailed unequal variance t-test were done with MetaboAnalyst 3.0 tool suite (Xia and Wishart 2016) with the p value threshold equal 0.05. Two-tailed Pearson correlation coefficients (r) between parasitaemia values and lipid levels, and between p(corr) vectors from the OPLS models were calculated with 95% confidence interval using the statistical package built into GraphPad Prism 6 software (San Diego, CA, U.S.A.). The Pearson correlations between lipid levels and clinical and personal data were calculated using in-house script written using the Anaconda Python distribution v. 3.5 (https://continuum.io). The Pearson correlation coefficients and the corresponding p-value against the null-hypothesis of no correlation were calculated using functions from the SciPy library (http://www.scipy.org/). The results were plotted using the Matplotlib library (http://matplotlib.org/).

## Results

### Plasma lipid levels correlated to certain clinical parameters

**Fig. 1:**
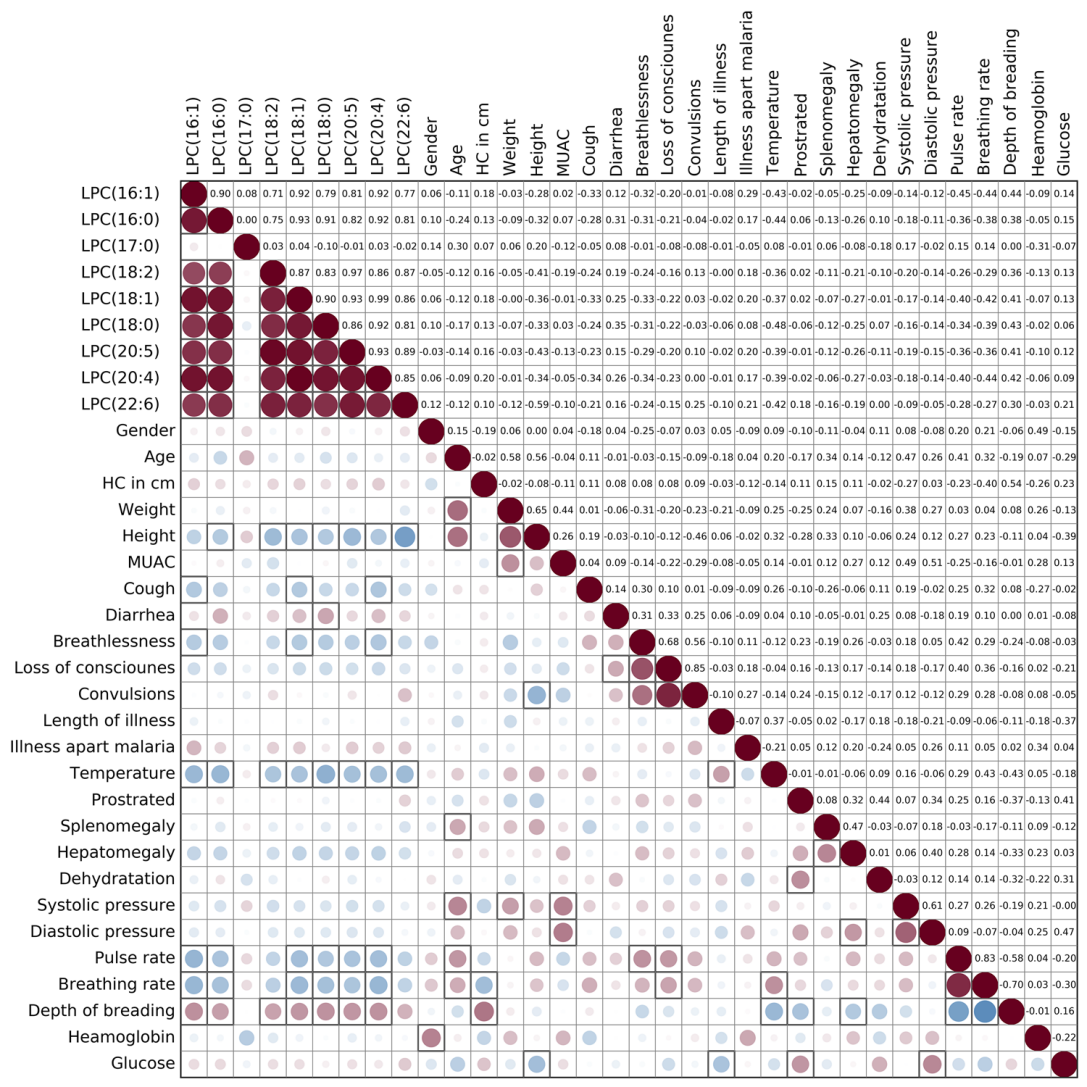
Correlation plot between clinical patient data and levels of lysophosphatidylcholines for infected individuals. The plot consists of two panels: (i) in the lower panel, *the color* and *size* of the *circles* correspond to the strength of the correlation, with increasing *circle size* and *color intensity* indicating increasing correlation; *shades of blue* are used for negative correlations and *shades of red* for positive correlations, *squares* indicate correlations that were statistically significant (p-value < 0.05), and (ii) the upper panel shows the corresponding Pearson’s correlation coefficients

We have correlated lipid levels in infected individuals to demographic and clinical characteristics of the study patients. The results are presented as Fig. 1 and Fig. S3–S6. A limited number of significant correlative relationships were found between certain lipids and clinical parameters. Even-chain length lysophosphatidylcholines (Fig. 1), were significantly (p < 0.05) positively correlated with diarrhea and depth of breathing and negatively correlated with height, pulse rate, breathing rate and temperature of the patients. The remaining lipids showed positive correlation with temperature, although significant only for selected phosphatidylcholines and sphingomyelins. Another visible trend was the positive correlation of TAGs with height and weight of the patients; longer chain TAGs were also positively correlated with age. Triglycerides with higher carbon content were negatively correlated with blood glucose levels of the patients.

### Separation of cases and controls as well as characterization of lipid distribution using PCA

**Fig. 2:**
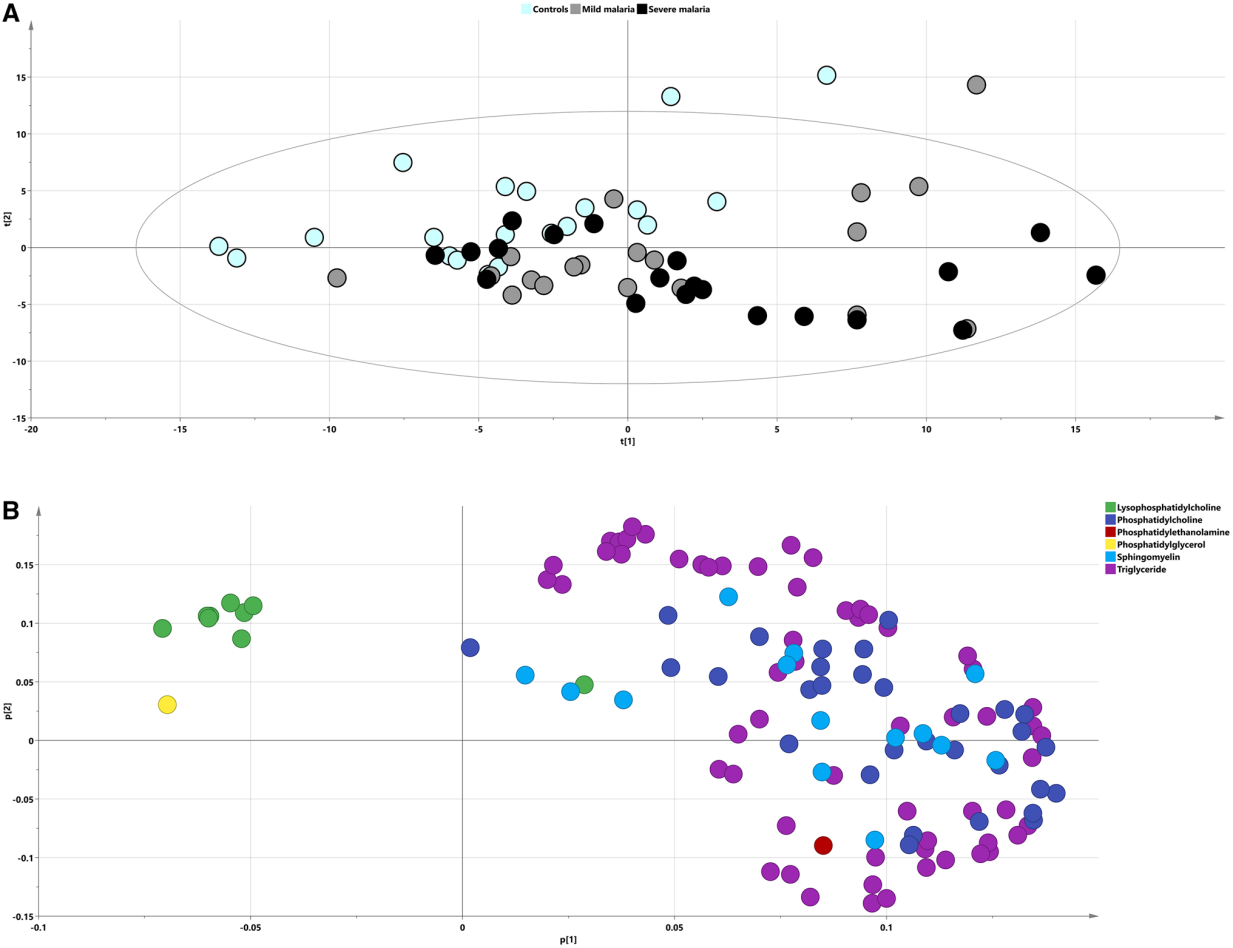
Lipidomic representation of patient groups. PCA score (**a**) and loading (**b**) plots on lipidomic data with samples colored according to their respective group: **a** *black dots* signify severe malaria samples, *gray dots* mild malaria and *blue* signify controls. **b** Lipid species on the loading plot are colored according to chemical classes. The separation of infected subjects and controls according to their lipidomic profiles is visualized in the plot; *x axis*—t[1] first score, *y axis*—t[2], second score

PCA was used to get an overview of all 117 identified lipid species from the 60 samples. One patient that belonged to the mild malaria group was found as an outlier on the PCA score plot and raw data revealed a lipid profile totally distinct from all other samples (data not shown). This sample was excluded from further analysis and a new PCA model was calculated (Fig. 2a; R2X(cum) = 0.803, Q2(cum) = 0.6, 6 components). Visible separation between infected and non-infected individuals could be seen along the two first components in the PCA score plot, with a shift from the upper left to the bottom right side of the PCA score plot that corresponded to the differences between controls and cases respectively. This trend was not skewed by the samples representing patients with concomitant infections. In order to support the statement we have calculated the ROC curves based on the first two PCA scores, with the AUROC values 0.760 for the first score and 0.808 for the second one (Fig. S7), demonstrating that there was sufficient (AUROC 0.6–0.7) to very good (AUROC 0.8–0.9) discrimination between studied groups of samples on the PCA score plot. Analysis of the corresponding loading plot (Fig. 2b) showed an increased abundance of selected compounds from the TAG group and decreased abundance of all detected even-chain LPCs in infected subjects as compared to the controls. No separation between mild and severe cases was visible in the plot.

### OPLS-DA case-control models show hierarchy of lipid responses

**Fig. 3:**
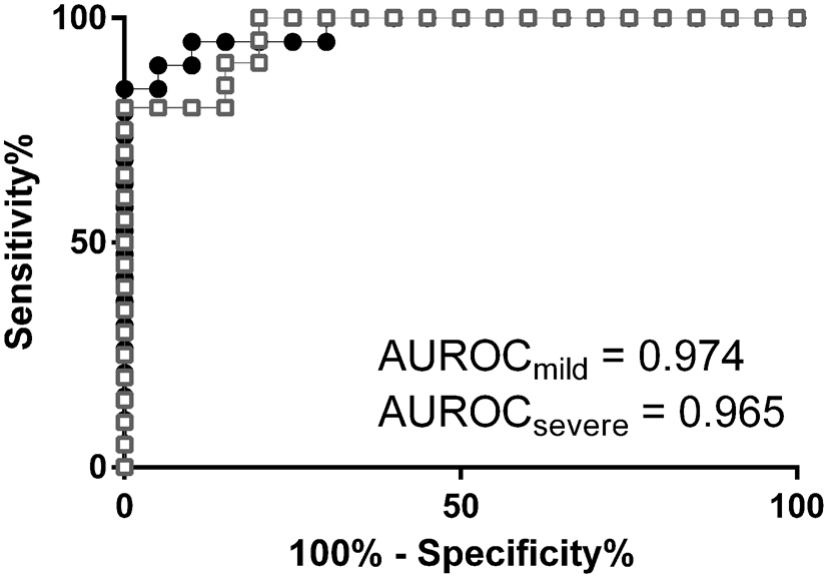
Lipid signature patterns as diagnostic separators. Diagnostic performance of lipid profile signature for severe malaria (*white squares*; AUROC = 0.9180 to 1.012 at 95% CI, p < 0.0001) and mild malaria (*black dots*; AUROC = 0.9329 to 1.015 at 95% CI, p < 0.0001); values of cross-validated predictive vector (t[1]cv) from the OPLS-DA models were taken for the ROC curves calculation

**Fig. 4:**
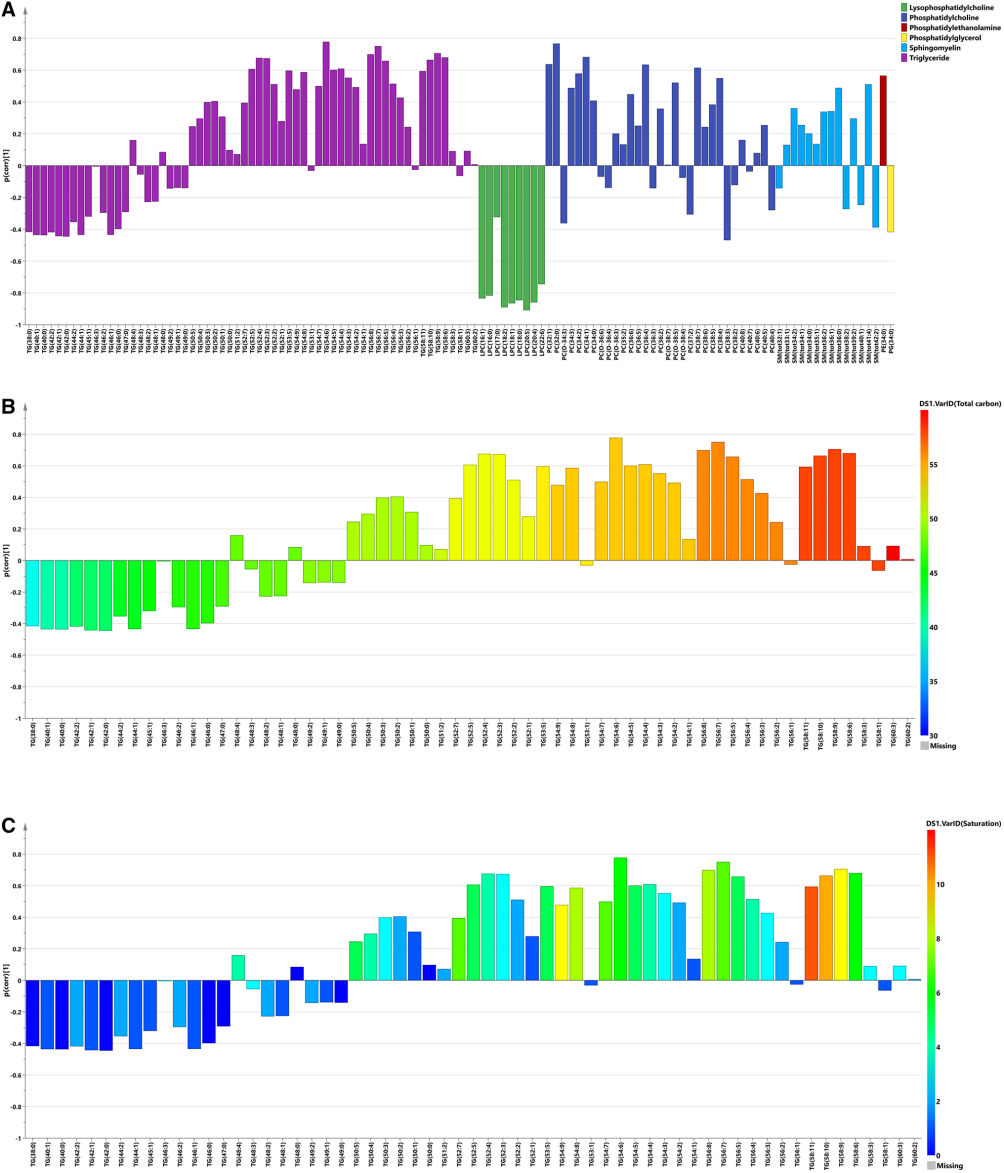
Lipid profile of infected individuals. **a** Predictive loading vector (p(corr)) from the OPLS-DA model between subjects infected with malaria and controls; lipid species are *colored* according to their chemical classes; p(corr) values indicate if the metabolite is in higher or lower levels in infected individuals compared with controls. **b** Predictive loading vector with p(corr) values for triglycerides colored according to total carbon content of the triglyceride molecule. **c** Predictive loading vector with p(corr) values for triglycerides colored according to total number of non-saturated locations in the triglyceride carbon chain

OPLS-DA was used to investigate further differences between the studied sample groups. We calculated OPLS-DA models comparing severe cases and controls (R2X(cum) = 0.55, Q2(cum) = 0.62, CV-ANOVA = 5.1 × 10^−7^ and 24% of variation in the data explained by predictive component) and mild cases and controls (R2X(cum) = 0.50, Q2(cum) = 0.72, CV-ANOVA = 5.8 × 10^−7^ and 19% of variation in the data explained by predictive component). The performance of the OPLS-DA models to separate classes of samples based on predicted cross-validated vector is presented in Fig. S8 as scatter plots and in Fig. 3 in form of ROC curves. P(corr) values from these models together with metabolites significant according to the model are listed in Table S4. It was not possible to obtain an OPLS-DA model between severe and mild cases, hence these two groups of samples could not be differentiated based on their lipid profiles. A shared and unique structure (SUS) plot analysis (Wiklund et al. 2008) of p(corr) vectors from severe versus controls and mild versus controls models revealed high correlation of their lipid profiles (r = 0.96, Fig. S9). This means that lipid profiling can be used to model differences between infected and not-infected individuals, but not between the malaria subgroups studied here. An OPLS-DA model between infected and not infected patients was constructed (R2X(cum) = 0.50, Q2(cum) = 0.59, CV-ANOVA = 6.0 × 10^−10^ and 21% of variation in the data explained by predictive component) to obtain a lipid profile related to the disease. The p(corr) values together with lipids significant for the model were compared with the previous models (Table S4). A combined lipid profile (p(corr)) where malaria cases and controls are compared is presented in Fig. 4. Significant metabolite fluctuations according to the jack-knifing confidence intervals are listed in Table S4. The entire profile was characterized by a majority of lipids showing elevated levels in infected individuals compared to controls, with exception of subgroup of TAGs and all LPCs. The main common feature of the TAGs subgroup compared to other species from this class was lower total length and a higher degree of saturation of the FA chains (average 46.3 vs. 53.8 carbon atoms and 1.1 versus 4.4 double bonds per molecule for TAGs with negative and positive p(corr) value respectively), which are to some extent correlated with each other (more possible double bonds can exist in the longer fatty acid chain). This means that TAGs carrying fatty acid chains with predominantly 0–2 unsaturation sites were present at lower levels in malaria cases, whereas TAGs with fatty acid chains with 3–12 unsaturation sites were higher in infected individuals compared to controls. The highly unsaturated TAGs with longer chain lengths that exhibited a high abundance in infected individuals were also the ones that were the most significant TAGs for separation between malaria infected individuals and controls as shown by both univariate (Tables S5, S6) and multivariate analysis (significance according to jack-knifing confidence intervals, Table S4).

### Univariate analysis of differences between the studied groups of samples

Significance of the differences between the means of the groups was tested with one-way ANOVA followed by post-hoc Tukey’s HSD test (Table S5). Student’s *t* test was used to compare means of compound levels in infected individuals versus controls (Table S6). Fifty-five compounds were found to be significant according to the one-way ANOVA (42 for mild-controls and severe-controls comparisons and 13 for severe-controls comparison only) and 62 for separation of all infected individuals from controls according to the *t* test (p value < 0.05). Univariate analysis confirmed findings from the multivariate modelling—a majority of lipids were present in significantly higher levels in the infected individuals, whereas LPCs were at significantly lower levels. Chain length and degree of saturation in TAGs were again identified as separators of infected individuals and controls. Triacylglycerides with longer fatty acid chains and higher degree of unsaturation were found at higher levels in infected individuals compared to controls.

### Lipid responses are inversely related to parasitaemia in comparison to case-controls

**Fig. 5:**
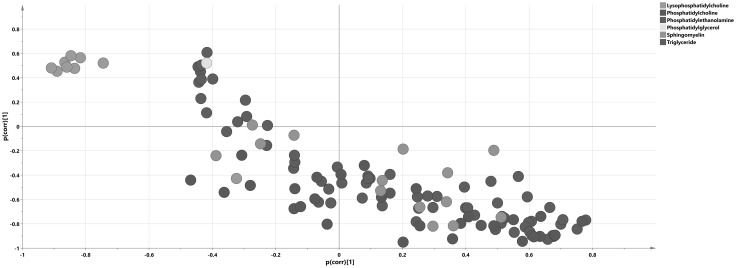
Global representation of lipids correlated to parasitemia. Shared and Unique Structure plot of correlation vectors (p(corr)) from the OPLS-DA infected individuals versus controls model (*X axis*) and OPLS-parasitaemia model for severe cases (*Y axis*). The lipids species are colored according to chemical class

Since parasitaemia is one way to estimate total parasite load we set out to investigate how this affects the plasma lipidome. In order to obtain a lipid profile connected to parasitaemia, an OPLS model was created with lipid levels as X matrix and parasitaemia values as Y. When we used all individuals with known parasitaemia scores (37 individuals from 41) the OPLS model, even though valid according to cross-validation, had a Q2 value below zero and could not be used to generate a parasitaemia-related lipid profile. We could however see that the majority of samples from the mild class were not well predicted by the model. We found a low span of values within this class of samples (0.37–4.02), and no model could be created for mild samples only. Improved linearity was observed for samples from the severe group (0.1–26.62 parasitaemia range) with the 37% of variation explained by the predictive component of the OPLS model. The lipid profiles showed a reduction of the majority of lipids with higher parasitaemia values, with the exception being even-chain LPCs and TAGs with lower total carbon content and higher degree of saturation (average 53.0 vs. 43.5 carbon atoms and 3.8 vs. 0.8 double bonds per molecule for TAGs with negative and positive p(corr) value respectively). The lipid profile correlated to increased parasitaemia values for severe cases is presented in Fig. S10 and summarized in Table S4. As such, the profile was reversely correlated (r = −0.86) with the profile obtained from the OPLS-DA model comparing the infected individuals and controls. This is visualized in a SUS plot in Fig. 5.

Univariate correlation analysis of each metabolite with parasitaemia revealed relationships that were significant (p-value < 0.05) according to the set threshold (Table S7). When all infected samples were considered, only phosphatidylethanolamine (34:0) demonstrated a significant negative correlation with parasitaemia. When the severe group only was considered, two lipid metabolites (TAG(42:2) and TAG(42:1)), were positively correlated with parasitaemia and 16 exhibited a negative correlation. The mild cases alone did not show any lipid species with significant correlation to parasitaemia.

## Discussion

The intricate fluctuation of metabolites in biological matrices during acute infectious stress has yet to be extensively investigated. We present herein an in depth cross-sectional study of the lipid profiles in pediatric malaria patients.

All detected even chain length LPCs were at lower levels in infected individuals compared to controls. The reduction was also body temperature dependent implying that, as seen in sepsis, the pyrogenic response plays a role in regulating the presence of LPCs in malaria. These species of lipids were also positively correlated with higher parasitaemia values. This means that even chain length LPCs were present at lower amounts in the malaria cases compared with the healthy controls. Contra intuitively however, LPC levels increased with parasitaemia. LPCs are plasma lipids that are created from phospatidylcholines by action of phospholipase A_2_ or by oxidation. They have been recognized as important cell signaling molecules, involved in wide range of physiological and pathophysiological processes (Fukushima et al. 2001; Birgbauer and Chun 2006). They are major components of oxidized low-density lipoproteins (oxLDL) and were previously described to be important inflammatory mediators with recognized effects in multiple immune cell types and hence connected to innate and also adaptive immune responses (Jackson et al. 2008; Kabarowski 2009). LPCs activate a number of secondary messengers by vast amount of signaling pathways as described in several reviews (Hla et al. 2001; Xu et al. 2003; Gardell et al. 2006; Torkhovskaya et al. 2007; zu Heringdorf and Jakobs 2007). LPC levels are markedly decreased in sepsis and their lowered levels show strong predictive power for sepsis-related mortality (Drobnik et al. 2003). In malarial infection intracellular activation of PLA-2 because of oxidative stress occurs in the infected red blood cells (Becker et al. 2004). This would lead to higher LPC levels in the infected erythrocytes. However, it is not the case extracellularly in the peripheral circulation. Administration of LPC in experimental sepsis has been shown to protect mice against sepsis-induced lethality (Yan et al. 2004), most probably by enhancing bactericidal activity of neutrophils. Immunosuppression in sepsis is believed to be the major contributing factor in sepsis-induced mortality (Hotchkiss and Karl 2003), and hence therapeutic action of LPC in sepsis can be attributed to its stimulatory effect on immune system (Kabarowski 2009). Since acute *P. falciparum* infection also induces an overwhelming acute phase response, it is possible that also in malaria administration of LPCs could have therapeutic effects and therefore be used to support treatment. An increase of LPC levels with higher parasitaemia values would on the other hand point towards an increased activation of immune system with higher amount of parasites in the blood. Our findings point to the important role of LPCs during malaria infection and hence confirm involvement of these molecules in the acute phase response in malaria.

LPC(17:0), the only odd chain LPC detected in our study, showed different behavior than other LPCs, with non-significant correlation with the disease and negative correlation to the parasitaemia values. Until recently it was believed that odd chain fatty acids in human plasma are mainly derived from food, but recent studies have showed that endogenous pathways of odd chain fatty acid biosynthesis can be active in humans (Jenkins et al. 2015). There are also data showing that the *P. falciparum* apicoplast fraction contains low levels of the odd-chain fatty acid, C17:0, which could be synthesized by either the type II FAS complex or by fatty acid elongases using propionyl-CoA instead of acetyl-CoA (Botté et al. 2013). More data is however needed to understand the role of odd chain fatty acids and their derivatives in the malaria infection.

We detected increased levels of a majority of all lipid species in infected individuals. This is in accordance with previous studies concerning the involvement of lipoproteins during the acute phase response. The response was most profound for TAGs with long-chain unsaturated FA, whereas shorter and more saturated species demonstrated a reverse pattern. This challenges our understanding of the acute phase response and the lipid release response. We show that in malaria infection the short chain TAGs are not included in the acute phase TAGs release. The TAGs response found in our study subjects has implications on cellular respiration and immunomodulation. A number of human and animal studies demonstrate that mitochondrial dysfunction significantly contribute to the development of sepsis-induced multi-organ failure, whereas a restoration of function is required for recovery (Haden et al. 2007; Harrois et al. 2009). Hecker et al. (Hecker et al. 2014) found that carnitine-independent catabolism of short chain FA through β-oxidation restores mitochondrial respiration to the same extent as long-chain FA. The authors suggest that short chain FA may be beneficial and also preferential in cellular respiration during stress. Our findings suggest that either these short FA are consumed at a higher rate or are sequestered by the host. Furthermore studies of lipid emulsions in cell systems show that FA imbalance affects immune-cell composition and function (Mayer et al. 2003a, b; Cury-Boaventura et al. 2006, 2008; Versleijen et al. 2008). Clinical trials have also indicated adverse effects of lipid emulsions rich in long-chain n-6 polyunsaturated fatty acids, at least in the critically ill patients. The supplementation of these FA resulted in an increased production of pro-inflammatory cytokines by mononuclear cells (Mayer et al. 2003a, b). We thus propose that the imbalance of long and short-chain FA contributes to the pathogenesis in acute malaria infection.

We hypothesize that the reversal of TAGs patterns in relationship to parasitaemia may be explained by the preferential parasite demand for long chain unsaturated TAGs in relation to short chain saturated TAGs. Our results clearly demonstrate that high parasitaemia scores correlates to low amounts of long unsaturated TAGs. The above profile appeared inverted when the cases and controls were compared. It is known since the 80s that *P. berghei* malarial infection results in an increase of the unsaturation level of triglycerides in the liver of mice (Deslauriers et al. 1988). This could speak for retention of unsaturated TAGs in the liver during infection and their subsequent release to the circulation. Although *Plasmodium* can synthesize a number of FAs that are subsequently incorporated into parasite membranes, it is highly dependent on the host for the acquisition of specific lipid molecules (Mi-ichi et al. 2006). Using host lipid intermediates (mainly FAs and LPCs), the parasite is capable of extensive synthesis and remodeling of its own phospholipids (Vial et al. 2003). Cholesterol, which is essential for parasite intra-erythrocytic growth (Bansal et al. 2005) and infection (Samuel et al. 2001), is also fully obtained from the host. Compared to the host cell membrane the parasite outer membrane is enriched in phosphatidylcholine and phosphatidylethanolamine and depleted in cholesterol and sphingomyelin. The cell membrane of erythrocytes infected with parasite also undergoes remodeling and exhibits a decrease in the level of polyunsaturated phospholipids (Vial et al. 2003). It has been shown that the different phases of the *P. falciparum* intra-erythrocytic life cycle are characterized by variations in lipid composition (Webster et al. 2009). Recent studies proved also that long chain unsaturated FAs are essential for parasite growth (Asahi et al. 2005; Ramakrishnan et al. 2015), some of which have to be synthesized by the parasites themselves (Ramakrishnan et al. 2012). The above relationship is actually beneficial to the parasite. Unsaturated FAs including C18:1, C18:2, and C20:4 have been shown to activate protein kinase C (PKC) dependently on or independently of Ca^2+^ and phospholipids (Murakami et al. 1986), and might be involved in sustaining parasite growth. Lipids are also necessary for the formation of hemozoin (Jackson et al. 2004; Pisciotta et al. 2007) and specifically unsaturated lipids were found to co-precipitate with ferriheme in the parasite’s acidic food vacuole and dissolve sufficient monomeric ferriheme to allow polymerization (Fitch et al. 1999). A reduction of levels of TAGs with long chain FAs in patients with high parasitaemia could also result from increased consumption of these FAs in the acyl-carnitine pathway indicating a paucity of the substrates needed for beta-oxidation in the high parasitaemic patients. In our study we could not find statistically significant differences between severe and mild cases in the lipidome. There may be several reasons for this; first and foremost we had no non-survivors in the cohort. Langley et al. also found no differences between sepsis, severe sepsis and septic shock survivors but found clear and reproducible differences between sepsis survivors and non-survivors (Langley et al. 2013). The nutritional status and proximity in time to feeding may also affect the outcome. However, one would expect statistically significant differences between the mild and severe cases since the severe cases have by definition difficulties in feeding, while the mild cases were less dehydrated and none of the patients with mild malaria were prostrated. Since our study is cross sectional, further investigation is needed to be able to fully appreciate the diversity of lipid responses in acute malaria infection.

## Conclusions

In conclusion, the key findings from the experiments performed concerning full plasma lipidomics in malaria patients can be summarized as follows: (a) the host lipid response to malaria infection is dynamic and multi-dimensional, with LPCs and TAGs levels responding inversely to disease and parasitaemia respectively; (b) the lipid perturbations are in themselves immunomodulatory and can affect energy turnover; (c) lipidomics may be used as tool to assess whole-body events in inflammation and acute infection by the use of plasma. It remains to see if the whole lipid acute phase response in other infections remain consistent with the response we have detailed herein.

## Electronic supplementary material

Below is the link to the electronic supplementary material.

Supplementary material 1 (PDF 3756 KB)

## Abbreviations

LCMS-QTOF: Liquid chromatography mass spectroscopy-quadrupole time of flight
SIRS: Systemic inflammatory response syndrome
TAG: Triacylglyceride
VLDL: Very low density lipoproteins
FA: Fatty acids
PCA: Principal component analysis
OPLS: Orthogonal projections to latent structures
LPC: Lysophosphatidylcholine
RSD: Relative standard deviation
