# Supplementary material for: Lipid response patterns in acute phase paediatric Plasmodium falciparum malaria
Source: Metabolomics. 2017 Feb 23;13(4):41. doi: 10.1007/s11306-017-1174-2 (PMC5323494; doi:10.1007/s11306-017-1174-2)
Supplement: Supplementary file 1 — Supplementary material 1 (PDF 3756 KB) [file 11306_2017_1174_MOESM1_ESM.pdf]

Lipid response patterns in acute phase paediatric *P. falciparum* malaria

SUPPLEMENTARY MATERIAL

Judy Orikiiriza[1,2,3,*], Izabella Surowiec[4*], Elisabeth Lindquist[5], Mari Bonde[6], Jimmy Magambo[3], Charles Muhinda[3,7], Sven Bergström[5,8,9], Johan Trygg[4+], Johan Normark[5,6,9,10+]

1. Infectious Diseases Institute, College of Health Sciences, Makerere University, P.O. Box 22418, Kampala, Uganda

2. Department of Immunology, Institute of Molecular Medicine, Trinity College Dublin, St. James's Hospital, Dublin 8, Ireland

3. Rwanda Military Hospital, P.O. Box: 3377, Kigali, Rwanda

4. Computational Life Science Cluster (CLiC), Department of Chemistry, Umeå University, 901 87 Umeå, Sweden

5. Department of Molecular Biology, Umeå University, 901 87 Umeå, Sweden

6. Department of Chemistry, Umeå University, 901 87, Umeå, Sweden

7. Department of Immmunology and Microbiology, School of Biomedical Sciences College of Health Sciences, Makerere University, P.O Box 7072, Kampala, Uganda

8. Laboratory for Molecular Infection Medicine Sweden (MIMS), 901 87 Umeå, Sweden

9. Umeå Center for Microbial Research, 901 87 Umeå, Sweden

10. Division of Infectious Diseases, Department Clinical Microbiology, Umeå University, 901 87 Umeå, Sweden

[*, +] These authors contributed equally to the work

Corresponding author: Johan Normark

Department of Molecular Biology, Umeå University, SE-901 87 Umeå, Tel: +46-(0)90 7850811, Fax:

+ 46-(0)90 772630, Email: johan.normark@ umu.se

Patient selection

A total of 690 patients, between six months and six years of age were enrolled from January 2011 to September 2013 at Nyagatare Hospital in Nyagatare district, Kiziguro and Ngarama Hospital in Gatsibo district or health centres in the catchment areas of these three hospitals in the Eastern Province of Rwanda. We applied a multivariate design approach [1] to select samples representative for the whole cohort of patients consisting from 221 severe malaria, 233 mild malaria and 236 controls. The multi-dimensional clinical data were used as basis for representative selection of the patients. PCA modelling was used to summarize clinical data into a low-dimensional hyper plane, which was visualized as a score scatter plot. Two factorial, full factorial design was applied to Principal Component Analysis (PCA) plots of two-component models with clinical data as X variables. In each of the four corners of the scatter plot, two samples (patients) were selected along with two center points. This procedure was repeated for each gender and each disease category, resulting in a total of 60 samples selected for further investigation. In this way we selected representative samples of the multivariate space (defined as samples and clinical variables related to them). Variables that were not numerical were transformed into discrete variables. Only variables that differed between samples and contained more than two values different from median were used. Patients presenting with jaundice and those with a known history of antimalarial treatment before sampling were excluded from the selection process. Age, height, weight and head circumference were highly correlated to each other and caused bias in the models, hence only age was included in the full factorial design selection of samples. This was also the case regarding systolic and diastolic blood pressure where only systolic pressure was included.

Lipidomics analysis

For lipidomics analysis, plasma was thawed on ice and 110 µL of extraction mixture (chloroform/methanol (2:1, v/v) with nine internal standards (PA(17:0/17:0), PG(17:0/17:0), PE(17:0/17:0), PG(17:0/0:0/0:0), DG(17:0/17:0/0:0), TAG(17:0/17:0/17:0), PC(17:0/0:0), PC(19:0/19:0) and TAG(17:0/17:0/17:0)-13C3; final concentration of the standards in the samples was from 1 to 20 ng/µL, was added to 20 µL of plasma; then extraction was carried out using a MM301 vibration Mill (Retsch GmbH & Co. KG, Haan, Germany) at a frequency of 30 Hz for 2 min. The samples were left in room temperature for 60 min and thereafter centrifuged at 14 000 rpm for 3 min at 4°C. A 50 µL aliquot of lower phase was transferred to LC vial, 70 µL of chloroform/methanol (2:1, v/v) mixture was added and samples were shaken briefly before being analysed with LCMS-QTOF.

One µL of each sample was injected into an Agilent UPLC system (Infinity 1290) equipped with a UPLC column (Acquity CSH, 2.1 x 50 mm, 1.7 µm C18 in combination with a 2.1 mm x 5 mm, 1.7 µm VanGuard CSH precolumn; Waters Corporation, Milford, MA, USA). The UPLC system was coupled to an Agilent 6540 iFunnel Jet stream electrospray ion source (Agilent Technologies, Santa Clara, CA, USA). Mobile phases used were 60:40 ACN:water + 10 mM ammonium formate + 0.1% formic Acid (A) and 89.1:10.5:0.4 IPA:ACN:water + 10 mM ammonium formate + 0.1% formic acid (B). The following gradient was used: 0.0 - 15 % B at a flow rate of 0.5 mL/min, then B was increased to 30 % during 1.2 min, then to 55% during 0.3 min and held at 55 % for 3.5 min, then to 72% during 2 min, to 85% during 2.5 min and to 100% during 0.5 min where was held at 0.5 ml/min flow rate for additional 2 minutes after which flow rate was increased to 5ml/min and kept for 0.5 min to wash the injection valve. No MS data were acquired during washing and equilibration steps. Column was equilibrated at 0.5 ml/min

[1] Eriksson, L., et al., Multi- and megavariate data analysis. Basic Principles and applications. Third ed. 2013, Umea: UMETRICS AB. ISBN: 978-91-973730-5-0.

flow rate with 15% B for 1.5 min before next injection was done. Column oven temperature was held at 65°C and samples were kept in auto-sampler at 10°C. The analysis was done in positive ion mode.

A reference interface was connected for accurate mass measurements; the reference ions purine (4 µM) and HP-0921 (Hexakis(1H, 1H, 3H-tetrafluoropropoxy)phosphazine) (1 µM) both purchased from Agilent Technologies (Santa Clara, CA, USA) were infused directly into the MS at a flow rate of 0.08 mL/min for internal calibration, and the monitored ions were purine m/z = 121.05 and HP-0921 m/z = 922.0098. The gas temperature was set to 300°C, the drying gas flow to 8 L/min and the nebulizer pressure 40 psi. The sheath gas temp was set to 350°C and the sheath gas flow to 11 L/min. The capillary voltage was set to 4000 V in positive ion mode. The nozzle voltage was 0 V. The fragmentor voltage was 100 V, the skimmer 45 V and the OCT 1 RF Vpp 750 V. The collision energy was set to 0 V. The m/z range was 70 - 1700, and data were collected in centroid mode with an acquisition rate of 4 scans/s.

Table S1. Clinical information for the control patients included in the study (*). In bold marked samples analyzed for endocannabinoids content (see Methods section).

Study code	Age	Gender	Blood pressure	HC in cm	Weight in kg	Height in cm	MUAC in cm	Temperature	Pulse rate	Breathing rate	Date of recruitment
**NYC-76**	1 YR 1 MON	M	90/70	46	8	76	15	36.7	112	24	17/08/2012
**NYC-112**	1 YR 1 MON	F	100/80	51	13	89	17	37.4	98	24	22/08/2012
**NYC-117**	5 YR	F	100/85	49	20	117	16	37.2	92	24	22/08/2012
**NYC-118**	2 YR	M	110/75	51	12	98	16	37.4	102	24	22/08/2012
**NYC-123**	2 YR 6 MON	F	120/85	49	14	93	17	37	94	20	22/08/2012
**NYC-141**	3 YR	M	118/69	51	13	90	16	36.4	99	25	06/12/2012
**NYC-144**	2 YR 11 MON	F	NA	51	13	91	16	36.4	107	26	06/12/2012
**NYC-150**	2 YR 6 MON	F	NA	47	13	89	17	36.9	106	24	07/12/2012
**NYC-161**	4YR	M	100/70	50.5	13	95	15	36.3	101	12	18/04/2013
**NYC-177**	2 YR 6 M	F	100/50	47	10	87	15	36.5	120	22	24/04/2013
**NYC-178**	5 YR	M	115/90	50	15	90	17	36.36	104	34	24/04/2013
**NYC-180**	2 YR	M	90/90	48	9	84	14	36.1	100	30	25/04/2013
**NYC-184**	3 YR 7M	M	100/70	51	15	100	16	36.96	50	24	25/05/2013
**NYC-189**	4 YR	F	115/70	48.5	15	106	15.5	36.5	94	18	26/04/2013
**NYC-214**	5 YR 3 M	M	122/96	52	21	115	16	36.9	102	26	08/07/2013
**NYC-215**	5 YR 6 M	F	124/98	52	18	105	15	36.6	84	24	07/08/2013
**NYC-216**	4YR	F	108/86	51	18	105	16	36.7	94	24	07/08/2013
**NYC-231**	3 YR 6 M	F	NA	51	16	99	17	37	NA	NA	23/12/2013
**NYC-232**	4 YR	M	100/80	53	17	102	19	37	89	21	23/12/2013
**NYC-249**	6M	M	100/70	49	19	118	16	36.8	84	19	01/05/2014

(*) All children except NYC-189 were vaccinated and had negative or unknown HIV serostatus., NA – not assessed; HC - head circumference; MUAC - mid-upper arm circumference

Table S2. Clinical information for the mild malaria patients included in the study. In bold marked samples analyzed for endocannabinoids content (see Methods section).

**Study code**	**Age (months)**	**Gender**	**Vaccination status**	**Blood pressure**	**HC in cm**	**Weight in kg**	**Height in cm**	**MUAC in cm**	**Temperature**	**Pulse rate**	**Breathing rate**	**Length of current illness in days**	**Parasitemia (%)**	**Other illness**	**Other symptoms**	**Other symptoms (2)**	**Treatment received before coming to hospital**	**Date of recruitment**
**NGM-23**	33	M	NA	NA	50	10	NA	16	38.5	72	28	3	1.25	URTI+ AWD	Waterly stool	*		29/04/2011
**NYM-33**	26	M	NA	100/60	50	17	89	16	38.6	72	20	4	4.02	Flu		*	Antipyretics, cough medicine	07/05/2011
**NYM-40**	23	F	NA	98/60	49	14	87	18	38.9	72	20	2	0.58		Nausea, loss of appetite		Antipyretics	12/05/2011
**NYM-47 ******	13	F	NA	97/55	46	12	71	14	38.9	72	20	3	0.37	Skin lesions	Shivering, vomiting	*		19/05/2011
**NYM-55**	53	M	NA	NA	42	18	100	NA	39	100	24	2	1.39			*		09/03/2012
**NYM-56**	60	M	NA	NA	54	19	117	NA	NA	98	22	NA	0.94		Vomiting	*, ^	Cotrimoxazol e	09/03/2012
**NYM-61**	45	M	NA	95/60	50.5	12	95	NA	NA	120	22	4	0.58			*		18/04/2012
**NYM-87**	60	F	NA	85/50	53.5	22	130	NA	39.54	130	36	3	1.9		Headache, anorexia	*		02/05/2012
**NYM-108**	48	F	NA	90/60	49	15	98	14	37.08	98	28	1	1.14					16/05/2012
**NYM-129**	49	M	NA	90/60	51	15	100	14	36.8	138	30	3	0.17		Abdominal pain, loss of appetite	*		27/06/2012
**NYM-142**	51	F	YES	110/80	53	17	100	15	39.1	130	26	1	3.7		Headache			04/07/2012
**NYM-147**	48	M	YES	100/50	49	11	91	13.5	36.7	120	26	1	1.38			*		06/07/2012
**NYM-151**	48	F	YES	110/80	48	15	96	17	36.3	95	20	2	NA		Headache			07/07/2012
**NYM-155**	39	M	YES	100/70	51.5	15	95	17	37.1	100	26	1	NA		Shivering, chills	*		09/07/2012
**NYM-160**	25	F	YES	85/60	48	10	80	14	37	104	24	2	0.89					11/07/2012
**NYM-183**	66	M	YES	90/55	52	18	109	15	37.5	118	20	1	2.34	Intestinal worms	Abdominal pain, headache	**, ^		02/08/2012
**NYM-196**	48	M	YES	110/79	49	17	94	16	38.5	98	21	1	NA			*		05/12/2012
**NYM-211**	62	F	YES	NA	50	14	104	15	36.59	100	22	3	1.32	Tonsilitis				09/12/2012
**NYM-215**	72	F	YES	115/80	50.5	20	106	17	38.3	106	28	1	1.17			*, **, ***, ~		17/12/2012
**NYM-229**	60	F	YES	120/70	5	18	113	16.5	39	170	60	2	1.3		Stomach ache		Anti-bacterial	05/03/2012

* - cough
** - diarrhea
*** - signs of dehydration
^ - splenomegaly
~ - dry mucus membrane
NA – not assessed
**** - patient excluded from the study
HC - head circumference
MUAC - mid-upper arm circumference

Table S3. Clinical information for the severe malaria patients included in the study. In bold marked samples analyzed for endocannabinoids content (see Methods section).

Study code	Age (months)	Gender	Vaccination status	Blood pressure	HC in cm	Weight in kg	Height in cm	MUAC in cm	Temperature	Pulse rate	Breathing rate	Blantyre coma scale	Length of current illness in days	Parasitemia (%)	Other illness	Other symptoms	Other symptoms (2)	Treatment received before coming to hospital	Date of recruitment	Severe malaria subgroup (*)
**NYS-33**	51	F	NA	90/60	45	12	100	14	40.09	162	52	5	2	0.4		Abdominal pain, headache	*, °°°, ***, °°, (s)		16/05/2012	RD
**NYA-69**	27	M	YES	90/60	48	11	86	14	38	118	26	5	3	6.58			°, ***, ~, (s)		12/07/2012	SA
**NYS-87**	36	F	YES		47	13	87	16	39.97	120	29	5	6	26.62		Prostation	**, °, ~, (s)		08/12/2012	RD
**NYS-106**	56	F	YES	100/74	50.5	13	44	14.2	36.69	102	26	5	1	9.88	Typhoid fever		°, ***, (n)		13/12/2012	HYP
**NYS-110**	18	F	YES		46	14		17.5	36.18	116	30	5	1	12.47			*, **, ~~, °, ~, °°, (n)		17/12/2012	HYP
**NYA-112**	48	M	YES	108/78	52	18	104	16	38.4	96	24	5	2	5.45	Traditional uvulectomy	Abdominal discomfort	°, (n)	Amoxycycline	18/12/2012	HYP
**NYS-117**	48	M	YES		50	18	103	16	39.8	110	30	5	2	15.02		Restlessnes, irritabilitys	°, °°, (s)		20/12/2012	HYP
**NYS-121**	29	M	YES		51	13	92	13.5	35.7	140	30	5	2	17.53		Vomiting	^^, ^^^, °, (n)		05/02/2013	FC
**NYS-125**	72	F	YES	120/50	50.5	15	107	14.5	39.4	190	60	5	3	5.95		Abdominal pain	*, **, ^^, ^^^, ~~(1), (s)	Ampicilin, paracetamol	05/03/2013	HG
**NYS-133**	53	M	YES	110/76	51	12	96	16	38.2	122	26	5	2	5.58		Vomiting	*, **, ^^, ~~(1), °, (n)	Traditional herbs	16/05/2013	PR
**NYS-138**	56	M	YES	106/92	50	15	101	17	37	156	36	5	1	2.7	Pneumonia	Lethargy, abdominal pain	*, ^^, °, H, ***, ~, (s)		20/05/2013	RD
**NYS-139**	24	M	YES	98/'74	50	11	80	16	38.99	164	46	5	1	10.6	Intestinal parasites	Vomiting	*, **, ^^, ^^^, °, H, (s)		20/05/2013	FC
**NYS-140**	55	M	YES	98/70	47	11	97	14	37.88	146	36	4	2	NA		Headache	*, ^^, ~~(3), W, (s)		20/05/2013	CM
**NYS-141**	62	F	NO	106/92	51	17	115	16		138	24	5	4	1.3	Bronchitis	Vomiting, shivers	^, H, (n)		22/05/2013	NUD
**NYS-150**	55	F	YES	118/98	50	15	106	15.5	38.5	164	34	5	2	3.2	Bronchitis	Flue, abdominal pain	*, °, (n)		23/05/2013	CM
**NYS-175**	72	M	YES	118/94	50	22	113	17	39.3	160	44	5	1	1.6		Headache, abdominal pain	*		13/07/2013	NUD
**NYS-190**	66	M	YES	110/90	52	19	111	16	37.3	116	40	5	1	7.4		Headache, abdominal pain	°°°, °, ^, H, J, W, °°, (s)		08/09/2013	HYP
**NYS-206**	64	F	YES	100/64	50	15	103	14	39.7	192	64	5	2	0.1		Shivers, vomiting	*, °, ***, ~, ~~~, (s)	Traditional herbs	18/9/2013	RD
**NYS-217**	64	F	NO	100/68	51	17	103	17	38.4	144	28	4	1	1.4		Headache, vomiting	°, ^, H, (s)		25/9/2013	PR
**NYS-92**	46	M	YES	100/80	47	12	100	16	39.34	108	26	5	4	6.64		Prostartion	°, ^, ***, ~, (s)		09/12/2012	HYP

* - cough; ** - diarrhea; *** - signs of dehydration; ^ - splenomegaly; ~ - dry mucus membrane; ^^ - breathlessness; ^^^ - loss of conscious; ~~ - convulsions (number); ~~~ - indrawing of intercostal spaces; ° - is patient prostrated; °° - nasal flaring () – depth of breading (n-normal, s-shallow); °°° - black urine; H- hepatomegaly; J – jaundice; W – wheezes; NA – not assessed; HC - head circumference; MUAC - mid-upper arm circumference;
CM – cerebral malaria; HYP – hyperparasitemia; RD – respiratory distress; SA – severe anemia; PR – prostration; FC - febrile convulsions; NUD - Severe Malaria Non Ultra Descriptus

Table S4. Lipids detected in plasma samples in the presented study and p(corr) values from the OPLS-DA models between studied groups of samples and OPLS-parasitaemia model for severe samples. In bold marked compounds significant according to the model, based on jack-knifing confidence intervals. Sign of p(corr) vector is given in relation to infected individuals (mild, severe or all cases) and higher parasitaemia values.

Compound	Molecular mass *	Molecular formula	Mild versus controls	Severe versus controls	Infected versus control	Parasitaemia in severe cases
TAG(38:0)	666.578	C41 H78 O6	-0.32	-0.38	-0.42	0.61
TAG(40:1)	692.596	C43 H80 O6	-0.27	-0.50	**-0.44**	0.23
TAG(40:0)	694.609	C43 H82 O6	-0.36	-0.39	-0.44	0.45
TAG(42:2)	718.613	C45 H82 O6	-0.33	-0.42	-0.42	0.11
TAG(42:1)	720.627	C45 H84 O6	-0.32	-0.46	-0.44	0.36
TAG(42:0)	722.641	C45 H86 O6	-0.34	-0.42	-0.45	0.49
TAG(44:2)	746.643	C47 H86 O6	-0.26	-0.36	-0.35	-0.04
TAG(44:1)	748.656	C47 H88 O6	-0.30	-0.45	-0.44	0.50
TAG(45:1)	762.674	C48 H90 O6	-0.17	-0.43	**-0.32**	0.04
TAG(46:3)	772.659	C49 H88 O6	0.00	-0.02	-0.01	-0.33
TAG(46:2)	774.674	C49 H90 O6	-0.19	-0.32	-0.30	0.21
TAG(46:1)	776.690	C49 H92 O6	-0.30	-0.45	-0.43	0.39
TAG(46:0)	778.706	C49 H94 O6	-0.23	-0.49	**-0.40**	0.39
TAG(47:0)	792.722	C50 H96 O6	-0.11	-0.47	-0.29	0.08
TAG(48:4)	798.675	C51 H90 O6	0.19	0.09	0.16	**-0.39**
TAG(48:3)	800.692	C51 H92 O6	-0.04	-0.06	-0.06	-0.45
TAG(48:2)	802.707	C51 H94 O6	-0.17	-0.23	-0.23	-0.15
TAG(48:1)	804.723	C51 H96 O6	-0.12	-0.26	-0.23	0.01
TAG(48:0)	806.738	C51 H98 O6	0.15	0.15	0.09	-0.46
TAG(49:2)	816.722	C52 H96 O6	0.03	-0.31	-0.14	-0.34
TAG(49:1)	818.737	C52 H98 O6	0.04	-0.33	-0.14	-0.29
TAG(49:0)	820.756	C52 H100 O6	0.04	-0.31	-0.14	-0.24
TAG(50:5)	824.691	C53 H92 O6	0.15	0.27	0.25	**-0.58**
TAG(50:4)	826.707	C53 H94 O6	0.25	0.31	0.30	**-0.67**
TAG(50:3)	828.723	C53 H96 O6	0.40	0.42	**0.40**	**-0.67**
TAG(50:2)	830.739	C53 H98 O6	0.41	0.44	**0.41**	**-0.67**
TAG(50:1)	832.755	C53 H100 O6	0.35	0.38	0.31	-0.57
TAG(50:0)	834.770	C53 H102 O6	0.20	0.12	0.10	-0.43
TAG(51:2)	844.754	C54 H100 O6	0.22	0.02	0.07	-0.59
TAG(52:7)	848.692	C55 H92 O6	**0.35**	0.44	0.40	**-0.50**
TAG(52:5)	852.723	C55 H96 O6	**0.58**	**0.68**	**0.61**	**-0.78**
TAG(52:4)	854.740	C55 H98 O6	**0.67**	**0.75**	**0.68**	**-0.90**
TAG(52:3)	856.755	C55 H100 O6	**0.69**	**0.74**	**0.67**	**-0.90**
TAG(52:2)	858.771	C55 H102 O6	0.53	**0.57**	**0.51**	**-0.80**
TAG(52:1)	860.786	C55 H104 O6	0.36	0.34	0.28	**-0.57**
TAG(53:5)	866.739	C56 H98 O6	**0.62**	**0.65**	**0.60**	**-0.79**
TAG(54:9)	872.686	C57 H92 O6	**0.51**	0.48	**0.48**	-0.45
TAG(54:8)	874.707	C57 H94 O6	**0.56**	**0.62**	**0.59**	**-0.83**
TAG(53:1)	874.800	C56 H106 O6	0.14	-0.15	-0.03	-0.51
TAG(54:7)	876.722	C57 H96 O6	0.45	**0.59**	**0.50**	**-0.63**
TAG(54:6)	878.739	C57 H98 O6	**0.80**	**0.82**	**0.78**	**-0.77**
TAG(54:5)	880.755	C57 H100 O6	**0.59**	**0.67**	**0.60**	**-0.87**
TAG(54:4)	882.771	C57 H102 O6	**0.63**	**0.66**	**0.61**	**-0.90**
TAG(54:3)	884.787	C57 H104 O6	**0.58**	**0.58**	**0.55**	**-0.87**
TAG(54:2)	886.802	C57 H106 O6	**0.53**	**0.55**	**0.49**	**-0.85**
TAG(54:1)	888.815	C57 H108 O6	0.23	0.15	0.14	**-0.65**
TAG(56:8)	902.737	C59 H98 O6	**0.74**	**0.74**	**0.70**	**-0.80**
TAG(56:7)	904.754	C59 H100 O6	**0.77**	**0.79**	**0.75**	**-0.84**
TAG(56:5)	908.785	C59 H104 O6	**0.66**	**0.72**	**0.66**	**-0.93**
TAG(56:4)	910.803	C59 H106 O6	**0.53**	**0.57**	**0.51**	**-0.74**

TAG(56:3)	912.818	C59 H108 O6	**0.44**	**0.47**	**0.43**	**-0.73**
TAG(56:2)	914.831	C59 H110 O6	0.18	0.32	0.24	**-0.78**
TAG(56:1)	916.843	C59 H112 O6	-0.06	0.03	-0.03	-0.63
TAG(58:11)	924.724	C61 H96 O6	**0.66**	**0.62**	**0.59**	-0.58
TAG(58:10)	926.738	C61 H98 O6	**0.73**	**0.69**	**0.66**	-0.66
TAG(58:9)	928.753	C61 H100 O6	**0.76**	**0.74**	**0.71**	**-0.77**
TAG(58:6)	934.804	C61 H106 O6	**0.65**	**0.75**	**0.68**	**-0.90**
TAG(58:3)	940.843	C61 H112 O6	-0.03	0.15	0.09	-0.41
TAG(58:1)	944.872	C61 H116 O6	-0.13	0.02	-0.06	**-0.62**
TAG(60:3)	968.876	C63 H116 O6	-0.02	0.15	0.09	**-0.43**
TAG(60:2)	970.890	C63 H118 O6	-0.15	0.10	0.01	**-0.47**
LPC(16:1)	493.318	C24 H48 N O7 P	**-0.77**	**-0.85**	**-0.83**	0.48
LPC(16:0)	495.334	C24 H50 N O7 P	**-0.78**	**-0.82**	**-0.82**	0.56
LPC(17:0)	509.349	C25 H52 N O7 P	-0.46	-0.28	-0.32	-0.43
LPC(18:2)	519.333	C26 H50 N O7 P	**-0.86**	**-0.88**	**-0.89**	0.45
LPC(18:1)	521.349	C26 H52 N O7 P	**-0.83**	**-0.86**	**-0.87**	0.53
LPC(18:0)	523.365	C26 H54 N O7 P	**-0.81**	**-0.85**	**-0.85**	0.58
LPC(20:5)	541.316	C28 H48 N O7 P	**-0.88**	**-0.90**	**-0.91**	0.48
LPC(20:4)	543.333	C28 H50 N O7 P	**-0.83**	**-0.86**	**-0.86**	0.49
LPC(22:6)	567.333	C30 H50 N O7 P	**-0.74**	**-0.72**	**-0.74**	0.52
PC(32:1)	731.549	C40 H78 N O8 P	**0.65**	**0.71**	**0.64**	**-0.74**
PC(32:0)	733.563	C40 H80 N O8 P	**0.78**	**0.83**	**0.77**	**-0.78**
PC(O-34:3)	741.567	C42 H80 N O7 P	-0.32	**-0.36**	-0.36	**-0.54**
PC(34:3)	755.548	C42 H78 N O8 P	0.43	**0.63**	**0.49**	**-0.82**
PC(34:2)	757.564	C42 H80 N O8 P	**0.54**	**0.64**	**0.58**	**-0.94**
PC(34:1)	759.580	C42 H82 N O8 P	**0.67**	**0.76**	**0.68**	**-0.89**
PC(34:0)	761.594	C42 H84 N O8 P	**0.40**	**0.48**	**0.41**	**-0.74**
PC(O-36:6)	763.548	C44 H78 N O7 P	-0.19	0.05	-0.07	-0.42
PC(O-36:4)	767.583	C44 H82 N O7 P	-0.07	-0.17	-0.14	-0.51
PC(O-36:3)	769.597	C44 H84 N O7 P	-0.02	**0.36**	0.20	**-0.95**
PC(35:2)	771.579	C43 H82 N O8 P	0.17	0.19	0.13	**-0.58**
PC(36:6)	777.535	C44 H76 N O8 P	0.41	**0.61**	**0.45**	**-0.81**
PC(36:5)	779.548	C44 H78 N O8 P	0.24	**0.42**	0.25	**-0.67**
PC(36:4)	781.564	C44 H80 N O8 P	**0.64**	**0.66**	**0.63**	**-0.90**
PC(36:3)	783.580	C44 H82 N O8 P	-0.14	-0.11	-0.14	**-0.68**
PC(36:2)	785.596	C44 H84 N O8 P	0.28	**0.41**	0.36	**-0.92**
PC(O-38:7)	789.567	C46 H80 N O7 P	0.01	0.07	0.01	-0.39
PC(O-38:5)	793.598	C46 H84 N O7 P	**0.53**	**0.52**	**0.52**	**-0.75**
PC(O-38:4)	795.614	C46 H86 N O7 P	0.00	-0.15	-0.08	-0.60
PC(37:2)	799.608	C45 H86 N O8 P	-0.28	-0.42	-0.31	-0.24
PC(38:7)	803.547	C46 H78 N O8 P	**0.60**	**0.64**	**0.62**	**-0.91**
PC(38:6)	805.564	C46 H80 N O8 P	0.26	0.24	0.24	-0.51
PC(38:5)	807.580	C46 H82 N O8 P	0.34	**0.46**	**0.38**	**-0.80**
PC(38:4)	809.596	C46 H84 N O8 P	**0.55**	**0.56**	**0.55**	**-0.77**
PC(38:3)	811.610	C46 H86 N O8 P	-0.43	**-0.45**	-0.47	-0.44
PC(38:2)	813.626	C46 H88 N O8 P	-0.14	-0.11	-0.12	**-0.66**
PC(40:8)	829.559	C48 H80 N O8 P	0.17	0.17	0.16	-0.55
PC(40:7)	831.577	C48 H82 N O8 P	0.06	-0.08	-0.04	**-0.80**
PC(40:6)	833.595	C48 H84 N O8 P	0.09	0.08	0.08	-0.32
PC(40:5)	835.610	C48 H86 N O8 P	0.18	0.30	0.25	**-0.82**
PC(40:4)	837.625	C48 H88 N O8 P	-0.26	-0.30	-0.28	-0.49
SM(tot32:1)	674.538	C37 H75 N2 O6 P	-0.09	-0.18	-0.14	-0.07
SM(tot33:1)	688.552	C38 H77 N2 O6 P	0.24	0.12	0.13	-0.53
SM(tot34:2)	700.552	C39 H77 N2 O6 P	0.27	**0.44**	0.36	**-0.81**
SM(tot34:1)	702.570	C39 H79 N2 O6 P	0.19	**0.36**	0.26	**-0.66**
SM(tot34:0)	704.584	C39 H81 N2 O6 P	0.21	0.29	0.20	-0.19
SM(tot35:1)	716.583	C40 H81 N2 O6 P	0.23	0.17	0.14	-0.45
SM(tot36:2)	728.584	C41 H81 N2 O6 P	0.27	**0.49**	**0.34**	**-0.62**
SM(tot36:1)	730.600	C41 H83 N2 O6 P	0.28	**0.48**	**0.34**	-0.38
SM(tot36:0)	732.613	C41 H85 N2 O6 P	**0.51**	**0.59**	**0.49**	-0.19

SM(tot38:2)	756.611	C43 H85 N2 O6 P	-0.32	-0.24	**-0.27**	0.01
SM(tot39:2)	770.634	C44 H87 N2 O6 P	**0.32**	0.43	0.30	**-0.82**
SM(tot40:1)	786.663	C45 H91 N2 O6 P	-0.12	**-0.30**	-0.25	-0.14
SM(tot41:4)	794.626	C46 H87 N2 O6 P	**0.49**	**0.54**	**0.51**	**-0.75**
SM(tot42:2)	812.679	C47 H93 N2 O6 P	-0.33	**-0.39**	**-0.39**	-0.24
PE(34:0)	719.548	C39 H78 N O8 P	**0.61**	**0.56**	**0.57**	-0.41
PG(34:0)	750.543	C40 H79 O10 P	-0.25	**-0.54**	-0.42	**0.52**

TAG – triacylglyceride, PC – phosphatidylcholine, LPC lysophosphatidylcholine, SM – sphingomyelin, PE – phosphatidylethanolamine, PS – phosphatidylserine, PG – phosphatidylglycerol, PI – phosphatidylinositol

* - determined experimentally;

Table S5. Compounds significant according to one-way ANOVA with post-hoc Tukey's HSD test showing which from the studied groups had significantly different means(p-values below 0.05) for the comparison of studied groups of samples.

Compound	One-way ANOVA p value	Tukey's HSD test
**TG(50:3)**	2.20E-02	Severe-Controls
**TG(50:2)**	9.21E-03	Mild-Controls; Severe-Controls
**TG(50:1)**	2.34E-02	Severe-Controls
**TG(52:7)**	2.63E-02	Severe-Controls
**TG(52:5)**	3.81E-03	Severe-Controls
**TG(52:4)**	5.37E-04	Mild-Controls; Severe-Controls
**TG(52:3)**	1.35E-04	Mild-Controls; Severe-Controls
**TG(52:2)**	1.85E-03	Mild-Controls; Severe-Controls
**TG(53:5)**	1.05E-02	Severe-Controls
**TG(54:9)**	3.91E-02	Mild-Controls; Severe-Controls
**TG(54:8)**	1.62E-03	Mild-Controls; Severe-Controls
**TG(54:6)**	7.61E-06	Mild-Controls; Severe-Controls
**TG(54:5)**	4.93E-03	Severe-Controls
**TG(54:4)**	1.61E-03	Mild-Controls; Severe-Controls
**TG(54:3)**	3.49E-03	Mild-Controls; Severe-Controls
**TG(54:2)**	2.93E-03	Mild-Controls; Severe-Controls
**TG(56:8)**	3.47E-04	Mild-Controls; Severe-Controls
**TG(56:7)**	4.87E-06	Mild-Controls; Severe-Controls
**TG(56:5)**	1.03E-04	Mild-Controls; Severe-Controls
**TG(56:4)**	8.12E-03	Severe-Controls
**TG(56:3)**	4.45E-02	Severe-Controls
**TG(58:11)**	2.10E-03	Mild-Controls; Severe-Controls
**TG(58:10)**	8.95E-04	Mild-Controls; Severe-Controls
**TG(58:9)**	1.80E-04	Mild-Controls; Severe-Controls
**TG(58:6)**	5.01E-05	Mild-Controls; Severe-Controls
**LPC(16:1)**	6.04E-09	Mild-Controls; Severe-Controls
**LPC(16:0)**	4.37E-07	Mild-Controls; Severe-Controls
**LPC(18:2)**	4.62E-12	Mild-Controls; Severe-Controls
**LPC(18:1)**	2.14E-09	Mild-Controls; Severe-Controls
**LPC(18:0)**	1.83E-10	Mild-Controls; Severe-Controls
**LPC(20:5)**	2.05E-12	Mild-Controls; Severe-Controls
**LPC(20:4)**	2.56E-09	Mild-Controls; Severe-Controls
**LPC(22:6)**	1.54E-04	Mild-Controls; Severe-Controls
**PC(32:1)**	1.68E-05	Mild-Controls; Severe-Controls
**PC(32:0)**	5.96E-08	Mild-Controls; Severe-Controls
**PC(O-34:3)**	5.14E-03	Mild-Controls; Severe-Controls
**PC(34:3)**	9.14E-04	Mild-Controls; Severe-Controls
**PC(34:2)**	1.19E-03	Mild-Controls; Severe-Controls
**PC(34:1)**	1.22E-06	Mild-Controls; Severe-Controls
**PC(34:0)**	9.33E-03	Mild-Controls; Severe-Controls
**PC(36:6)**	2.17E-03	Mild-Controls; Severe-Controls
**PC(36:5)**	4.97E-02	Severe-Controls
**PC(36:4)**	9.81E-05	Mild-Controls; Severe-Controls
**PC(O-38:5)**	1.14E-02	Mild-Controls; Severe-Controls
**PC(38:7)**	7.88E-05	Mild-Controls; Severe-Controls
**PC(38:5)**	7.88E-03	Mild-Controls; Severe-Controls
**PC(38:4)**	8.95E-03	Mild-Controls; Severe-Controls
**PC(38:3)**	7.65E-03	Mild-Controls; Severe-Controls
**SM(tot36:2)**	2.54E-02	Severe-Controls
**SM(tot36:1)**	6.06E-03	Severe-Controls
**SM(tot36:0)**	4.95E-04	Mild-Controls; Severe-Controls
**SM(tot39:2)**	3.39E-02	Severe-Controls
**SM(tot41:4)**	1.76E-02	Severe-Controls
**SM(tot42:2)**	1.03E-02	Mild-Controls; Severe-Controls
**PE(34:0)**	2.30E-04	Mild-Controls; Severe-Controls

**TG(50:3)**	2.20E-02	Severe-Controls
**TG(50:2)**	9.21E-03	Mild-Controls; Severe-Controls
**TG(50:1)**	2.34E-02	Severe-Controls
**TG(52:7)**	2.63E-02	Severe-Controls
**TG(52:5)**	3.81E-03	Severe-Controls
**TG(52:4)**	5.37E-04	Mild-Controls; Severe-Controls
**TG(52:3)**	1.35E-04	Mild-Controls; Severe-Controls
**TG(52:2)**	1.85E-03	Mild-Controls; Severe-Controls
**TG(53:5)**	1.05E-02	Severe-Controls

Table S6. P-values and fold changes for the compounds significant according to t-test (p-values in t-test below 0.05) for the comparison between infected individuals and controls.

**Compound**	**p-value**	**Fold change**
**TG(50:3)**	6.05E-03	0.70
**TG(50:2)**	1.39E-03	0.68
**TG(50:1)**	1.41E-03	0.70
**TG(52:7)**	3.46E-03	0.61
**TG(52:5)**	2.11E-04	0.56
**TG(52:4)**	5.11E-06	0.56
**TG(52:3)**	8.02E-07	0.59
**TG(52:2)**	1.47E-04	0.67
**TG(52:1)**	6.28E-03	0.69
**TG(53:5)**	4.58E-04	0.67
**TG(54:9)**	1.34E-03	0.60
**TG(54:8)**	1.33E-04	0.47
**TG(54:7)**	1.59E-02	0.58
**TG(54:6)**	1.52E-08	0.32
**TG(54:5)**	2.67E-04	0.61
**TG(54:4)**	7.00E-05	0.64
**TG(54:3)**	2.76E-04	0.68
**TG(54:2)**	1.31E-04	0.60
**TG(56:8)**	2.82E-06	0.43
**TG(56:7)**	1.54E-08	0.43
**TG(56:5)**	1.40E-06	0.53
**TG(56:4)**	1.54E-03	0.70
**TG(56:3)**	8.43E-03	0.68
**TG(58:11)**	1.72E-05	0.44
**TG(58:10)**	5.48E-06	0.39
**TG(58:9)**	5.92E-07	0.37
**TG(58:6)**	2.56E-06	0.57
**LPC(16:1)**	2.75E-07	2.74
**LPC(16:0)**	1.01E-05	3.04
**LPC(17:0)**	4.83E-02	1.02
**LPC(18:2)**	2.99E-08	4.30
**LPC(18:1)**	1.74E-07	3.07
**LPC(18:0)**	6.19E-08	3.72
**LPC(20:5)**	6.66E-09	3.72
**LPC(20:4)**	7.81E-08	2.64
**LPC(22:6)**	3.36E-05	2.38
**PC(32:1)**	4.96E-08	0.32
**PC(32:0)**	1.07E-10	0.36
**PC(O-34:3)**	8.95E-03	1.38
**PC(34:3)**	5.03E-05	0.60
**PC(34:2)**	7.04E-05	0.63
**PC(34:1)**	5.75E-09	0.46
**PC(34:0)**	3.79E-04	0.70

**PC(36:6)**	8.71E-05	0.64
**PC(36:5)**	4.39E-03	0.66
**PC(36:4)**	7.83E-06	0.62
**PC(O-38:5)**	1.71E-03	0.69
**PC(37:2)**	1.96E-02	1.46
**PC(38:7)**	1.78E-05	0.66
**PC(38:5)**	2.11E-03	0.74
**PC(38:4)**	9.31E-04	0.68
**PC(38:3)**	8.05E-03	1.39
**SM(tot34:0)**	3.42E-02	0.81
**SM(tot35:1)**	2.06E-02	0.78
**SM(tot36:2)**	6.33E-03	0.74
**SM(tot36:1)**	1.45E-03	0.70
**SM(tot36:0)**	3.84E-05	0.51
**SM(tot38:2)**	3.63E-02	1.30
**SM(tot39:2)**	4.01E-03	0.63
**SM(tot41:4)**	2.09E-03	0.67
**SM(tot42:2)**	9.25E-03	1.28
**PE(34:0)**	7.96E-05	0.97

Table S7. Results of the analysis of correlation between lipid species and parasitaemia.

**Compound**	**Pearson correlation coefficient for all samples**	**P-value for the Pearson correlation coefficient for all samples**	**Pearson correlation coefficient for severe cases only**	**P-value for the Pearson correlation coefficient for severe cases only**
Positive correlation with parasitaemia				
TAG(42:2)			0.541	0.017
TAG(42:1)			0.459	0.048
Negative correlation with parasitaemia				
TAG(52:5)			-0.467	0.044
TAG(52:4)			-0.553	0.014
TAG(52:3)			-0.533	0.019
TAG(53:5)			-0.534	0.018
TAG(56:5)			-0.493	0.032
PC(32:0)			-0.476	0.040
PC(34:2)			-0.460	0.048
PC(34:1)			-0.474	0.040
PC(O-36:3)			-0.518	0.023
PC(36:4)			-0.572	0.012
PC(O-38:5)			-0.533	0.019
PC(38:7)			-0.570	0.011
PC(38:4)			-0.523	0.022
PC(38:2)			-0.536	0.018
SM(tot41:4)			-0.510	0.026
PE(34:0)	-0.361	0.033	-0.531	0.019

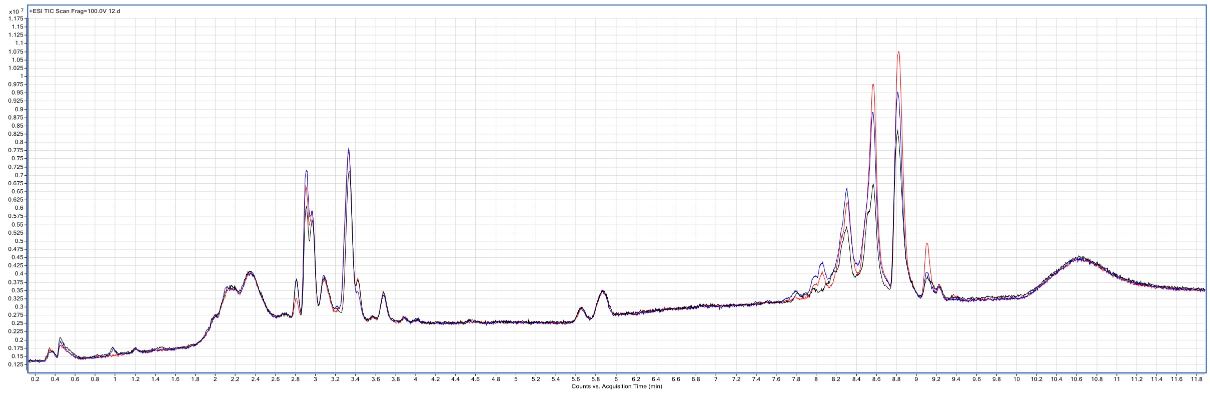

Fig. S1. Example TICs from the controls (black), mild malaria (blue) and severe malaria (red) groups.

A)

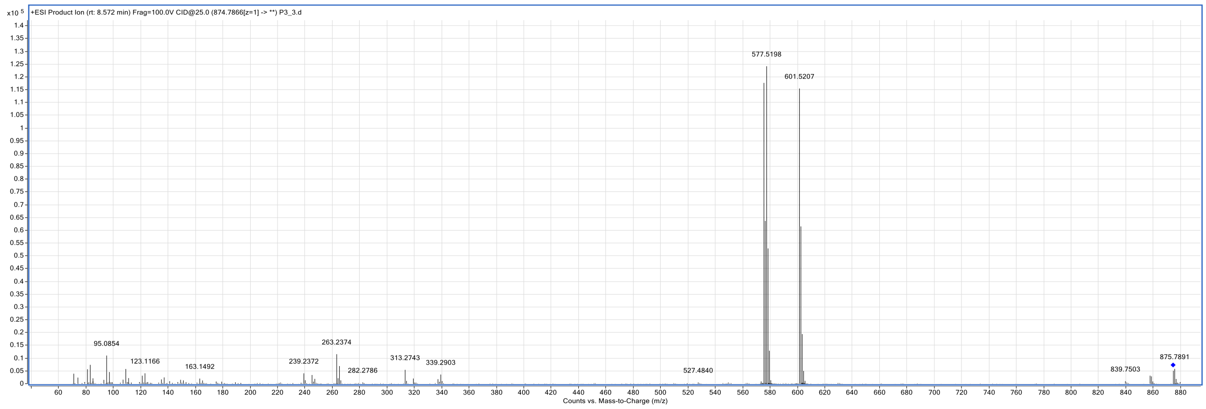
x10 5
+ESI Product Ion (rt: 8.572 min) Frag=100.0V CID@25.0 (874.7866[z=1] -> **) P3_3.d
577.5198
601.5207
95.0854
123.1166
163.1492
239.2372
263.2374
282.2786
313.2743
339.2903
527.4840
839.7503
875.7891
Counts vs. Mass-to-Charge (m/z)

B.

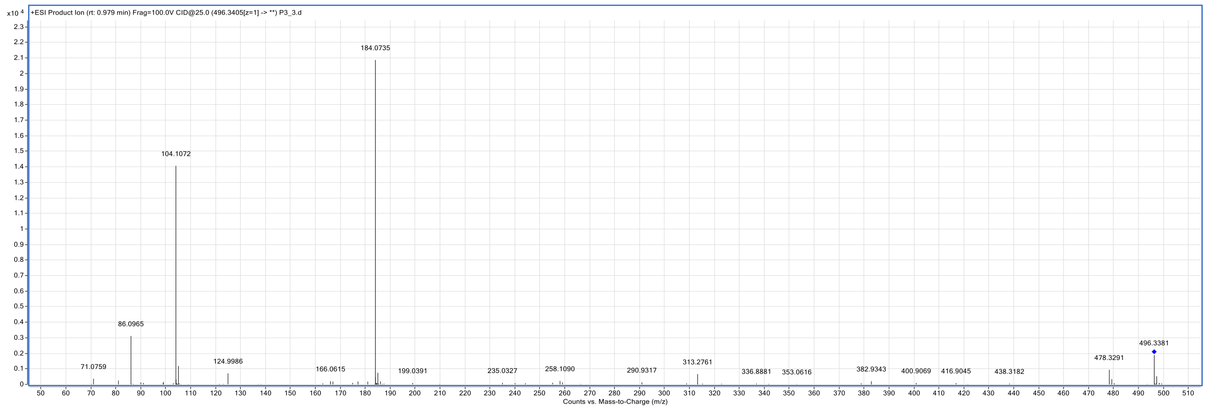
x10 4
+ESI Product Ion (rt: 0.979 min) Frag=100.0V CID@25.0 (496.3405[z=1] -> **) P3_3.d
184.0735
104.1072
86.0965
71.0759
124.9986
166.0615
199.0391
235.0327
258.1090
290.9317
313.2761
336.8881
353.0616
382.9343
400.9069
416.9045
438.3182
478.3291
496.3381
Counts vs. Mass-to-Charge (m/z)

C.

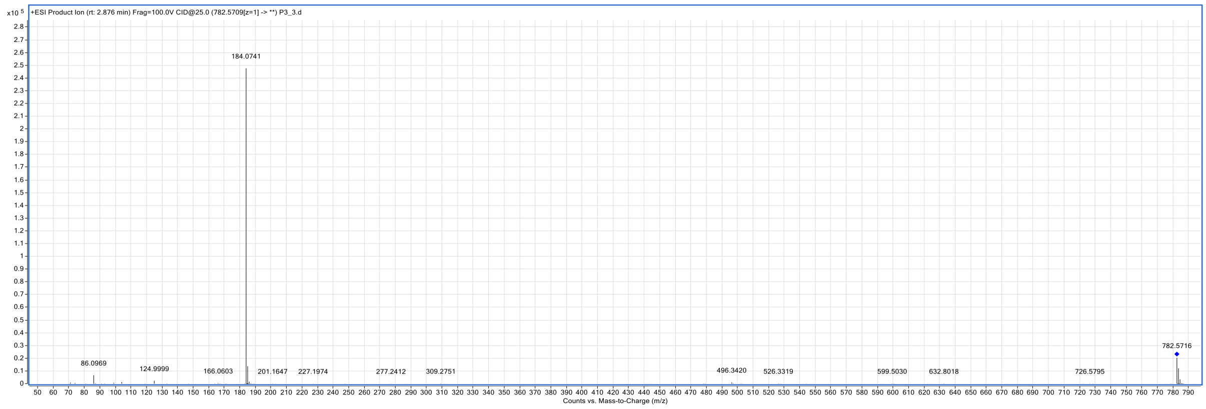
x10 5
+ESI Product Ion (rt: 2.876 min) Frag=100.0V CID@25.0 (782.5709[z=1] -> **) P3_3.d
184.0741
86.0969
124.9999
166.0603
201.1647
227.1974
277.2412
309.2751
496.3420
526.3319
599.5030
632.8018
726.5795
782.5716
Counts vs. Mass-to-Charge (m/z)

D.

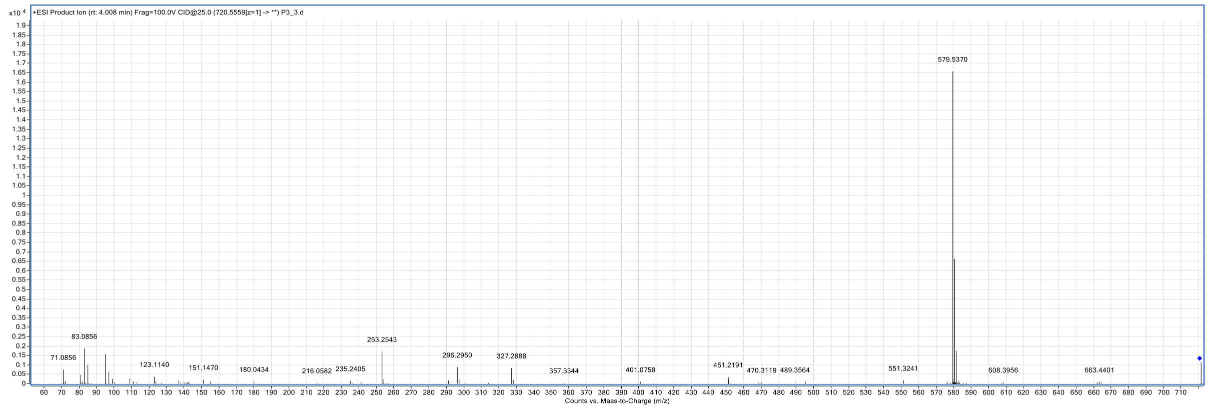

E.

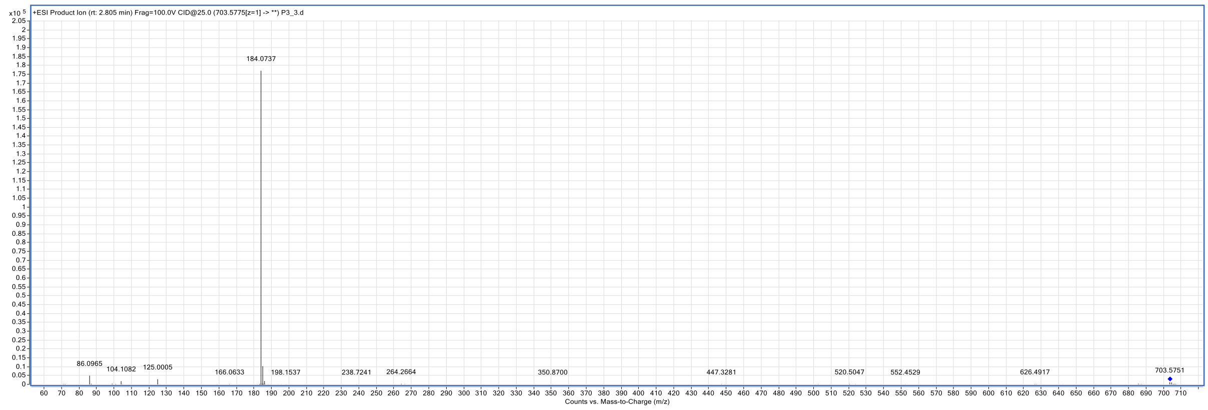

Fig. S2. MS/MS spectra at collision energy 25V of the example molecules from the main detected classes of compounds: A. $[TAG(52:3)+NH_4]^+$ (m/z=874.7894) with characteristic ions: m/z=239, m/z=263 and m/z=265 corresponding to the fatty acid groups and m/z=575.5052, m/z=577.5198 and m/z=601.5207 corresponding to the products of the loss of the fatty acid ammonium salts from the $[TAG(52:3)+NH4]^+$ ion; B. $[LPC(16:0)+H]^+$ (m/z=496.3418) with characteristic ions: m/z=184, m/z=104 and m/z=86 corresponding to the phosphocholine group and its daughter ions respectively; C. $[PC(36:4)+H]^+$ (m/z=782.5718) with characteristic phosphocholine group ion m/z=184; D. $[PE(34:0)+H]^+$ (m/z=720.5558) with characteristic neutral loss 141; E. $[SM(tot34:1)+H]^+$ (m/z=703.5778) with characteristic phosphocholine group ion m/z=184.

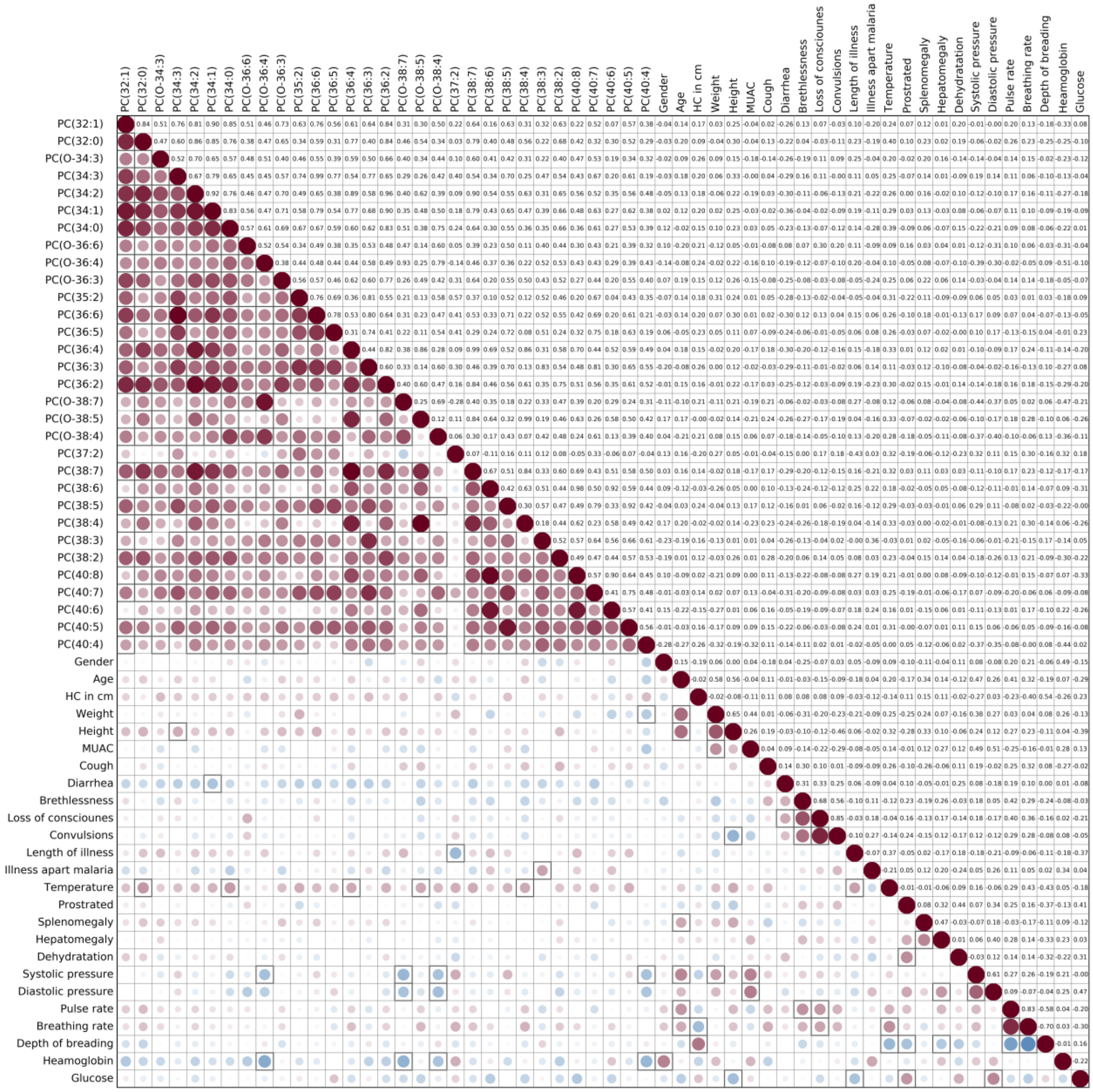

Fig. S3. Plot showing correlation between clinical and personal patient data and phosphatidylcholines for infected individuals. The plot consists of 2 panels: (i) in the lower panel, the color and size of the circles correspond to the strength of the correlation, with increasing circle size and color intensity indicating increasing correlation; shades of blue are used for negative correlations and shades of red for positive correlations, squares indicate correlations that were statistically significant (p-value < 0.05), and (ii) the upper panel shows the corresponding Pearson's correlation coefficients.

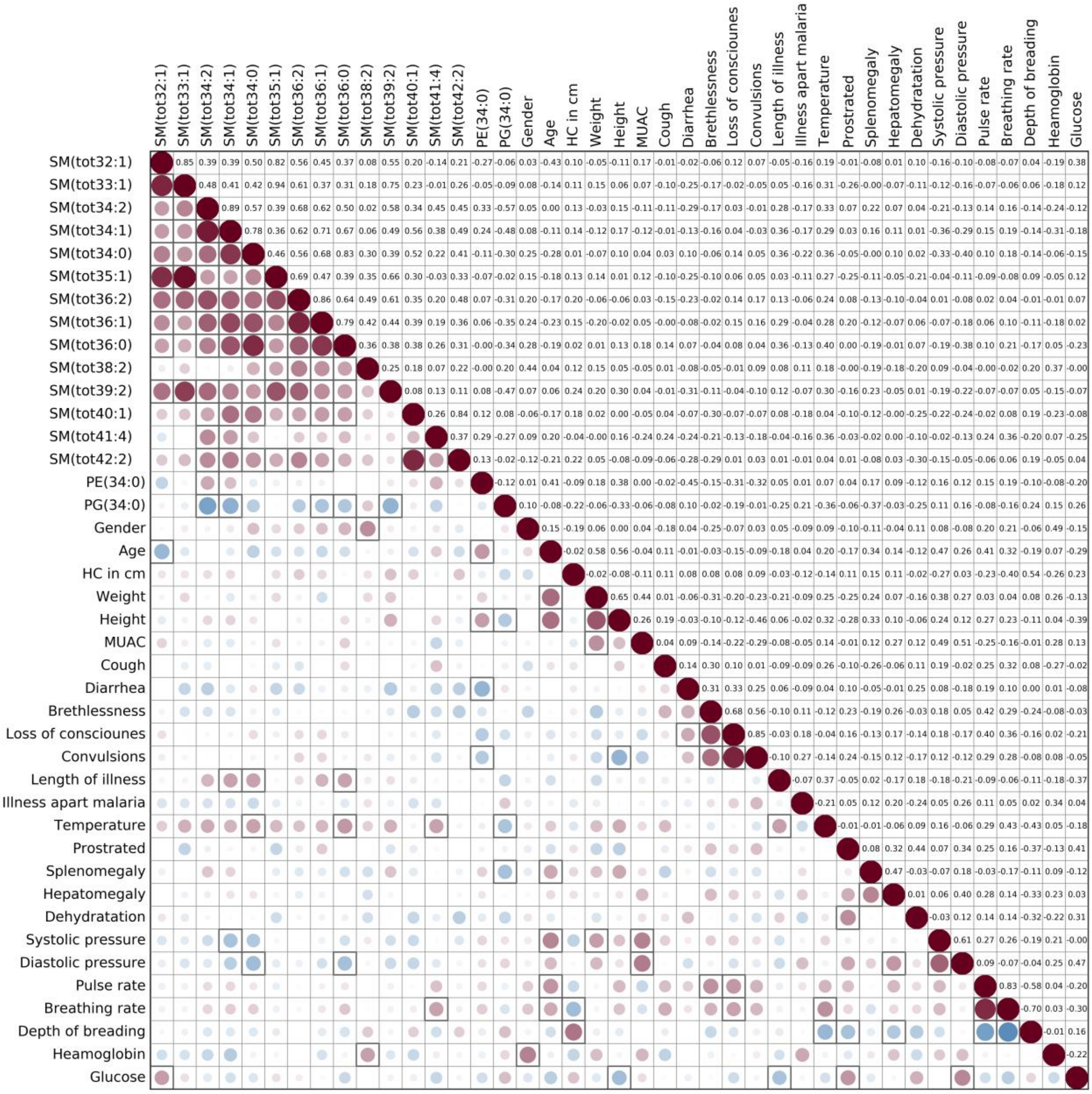

Fig. S4. Plot showing correlation between clinical and personal patient data and detected sphingomyelins and other lipids for infected individuals. The plot consists of 2 panels: (i) in the lower panel, the color and size of the circles correspond to the strength of the correlation, with increasing circle size and color intensity indicating increasing correlation; shades of blue are used for negative correlations and shades of red for positive correlations, squares indicate correlations that were statistically significant (p-value < 0.05), and (ii) the upper panel shows the corresponding Pearson's correlation coefficients.

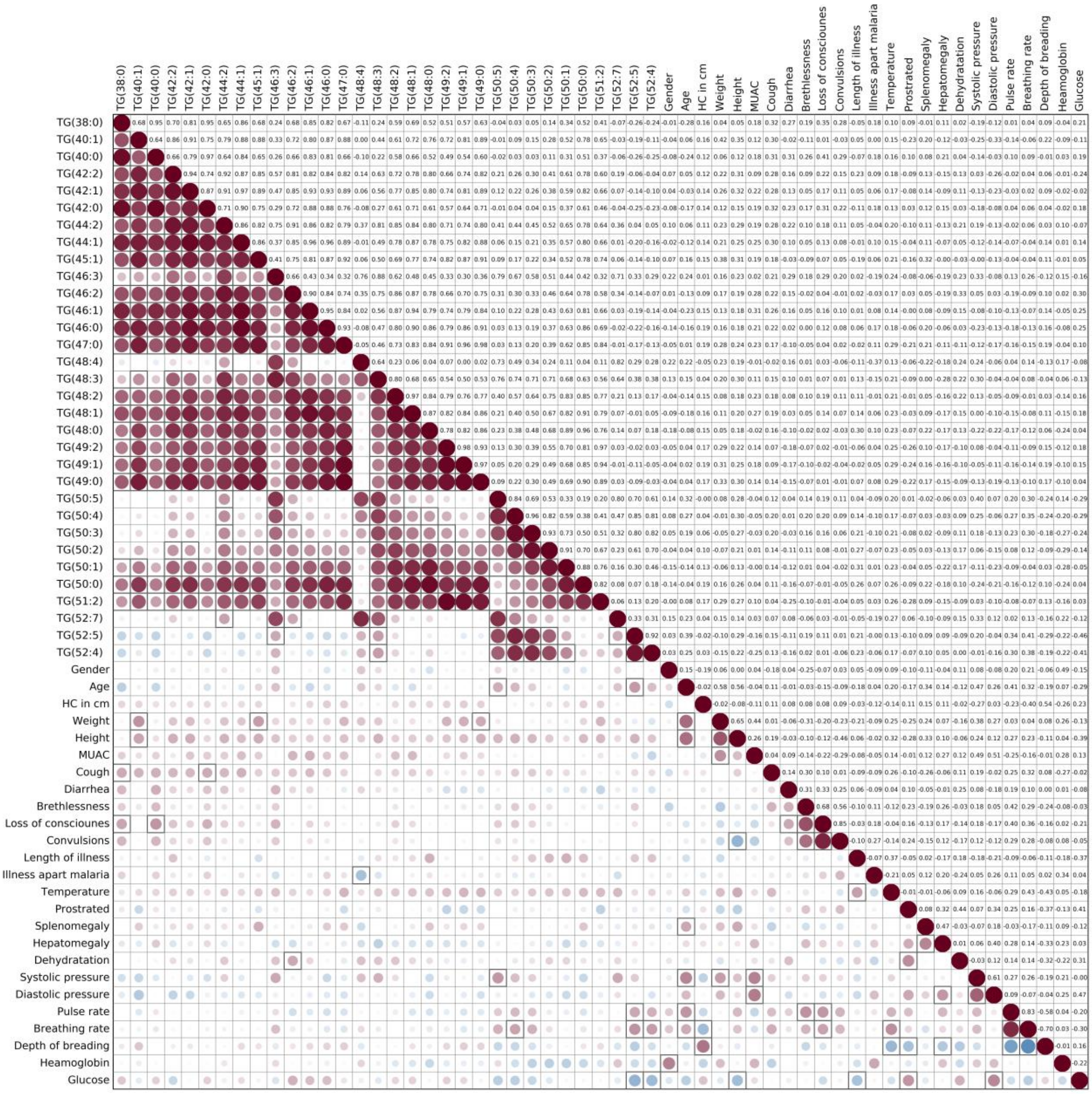

Fig. S5. Plot showing correlation between clinical and personal patient data and triacylglycerols with lower total carbon content for infected individuals. The plot consists of 2 panels: (i) in the lower panel, the color and size of the circles correspond to the strength of the correlation, with increasing circle size and color intensity indicating increasing correlation; shades of blue are used for negative correlations and shades of red for positive correlations, squares indicate correlations that were statistically significant (p-value < 0.05), and (ii) the upper panel shows the corresponding Pearson's correlation coefficients.

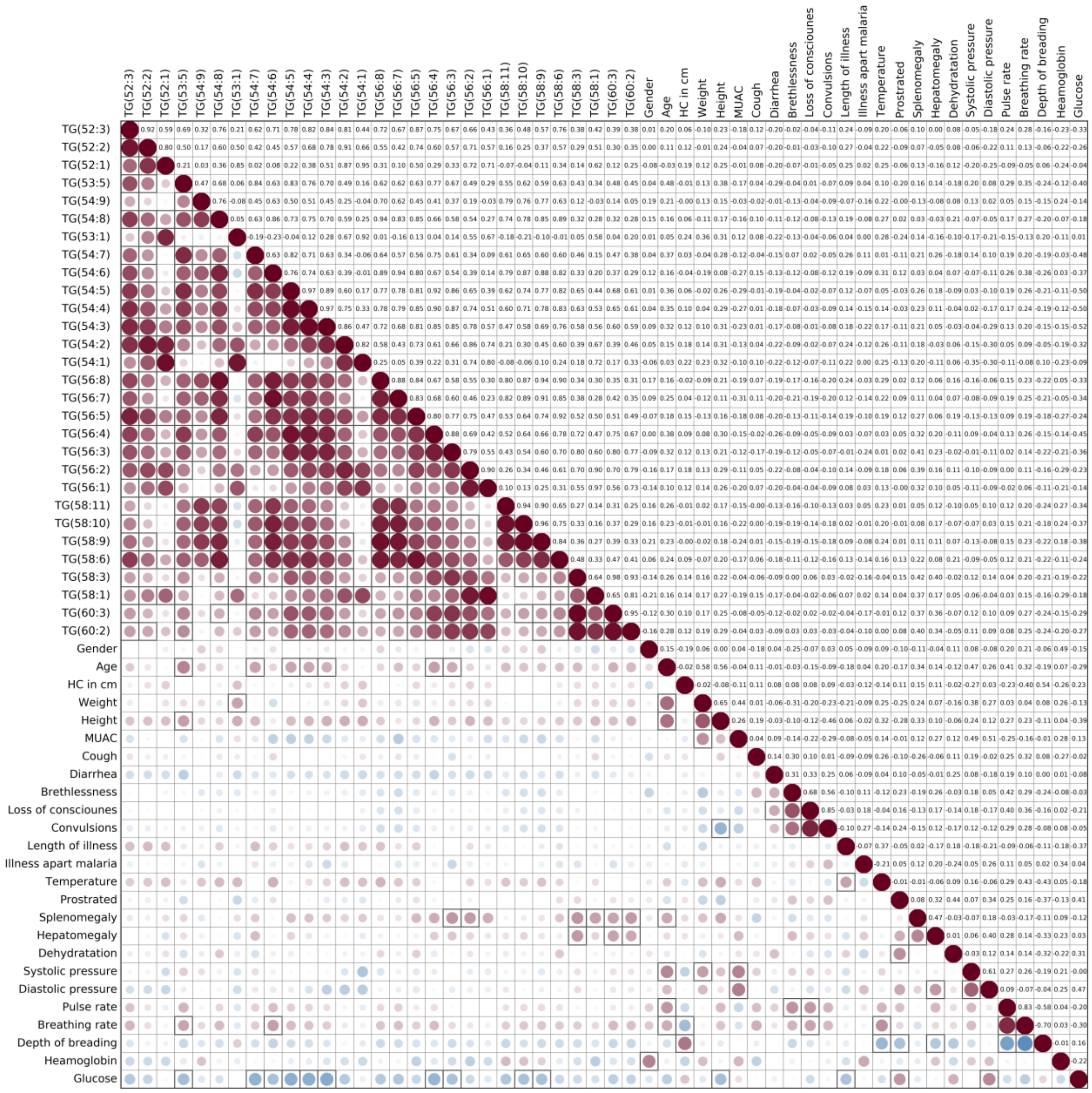

Fig. S6. Plot showing correlation between clinical and personal patient data and triacylglycerols with higher total carbon content for infected individuals. The plot consists of 2 panels: (i) in the lower panel, the color and size of the circles correspond to the strength of the correlation, with increasing circle size and color intensity indicating increasing correlation; shades of blue are used for negative correlations and shades of red for positive correlations, squares indicate correlations that were statistically significant (p-value < 0.05), and (ii) the upper panel shows the corresponding Pearson's correlation coefficients.

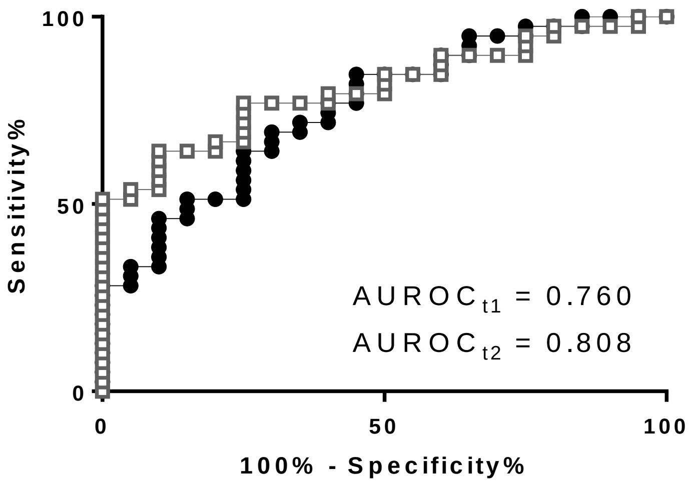

Fig. S7. Diagnostic performance of the PCA analysis; values of cross-validated score vectors from the PCA model on all samples and detected lipid species were taken for the ROC curves calculation: t1 - black dots; AUROC = 0.6344 to 0.8861 at 95% CI, p = 0 0012; t2 – white squares; AUROC = 0.6999 to 0.9155 at 95% CI, p = 0.0001);

A.

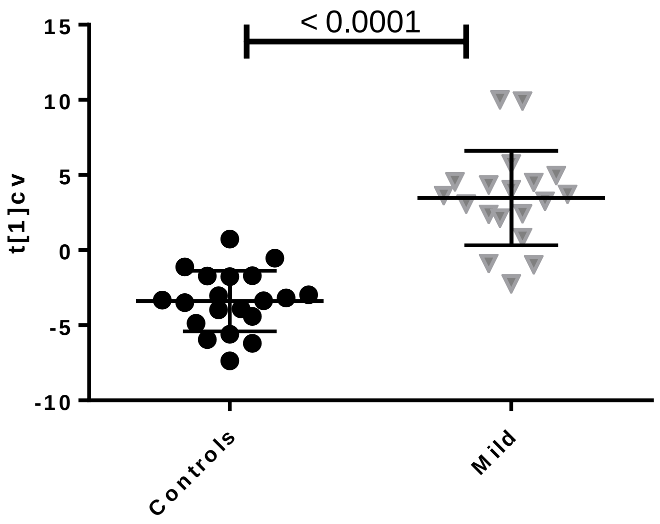

B.

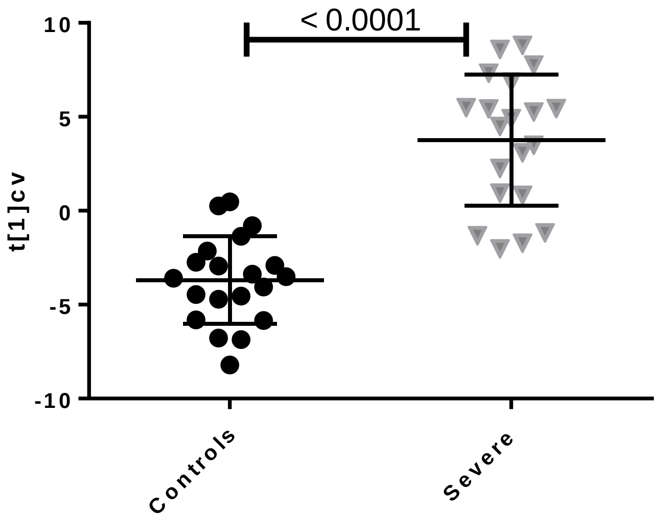

Fig. S8. Separation score plots of patient groups. OPLS-DA score values of the first predictive component (t[1]cv) showing the separation between A - mild malaria and controls ($p < 0.0001$), B – severe cases and controls ($p < 0.0001$).

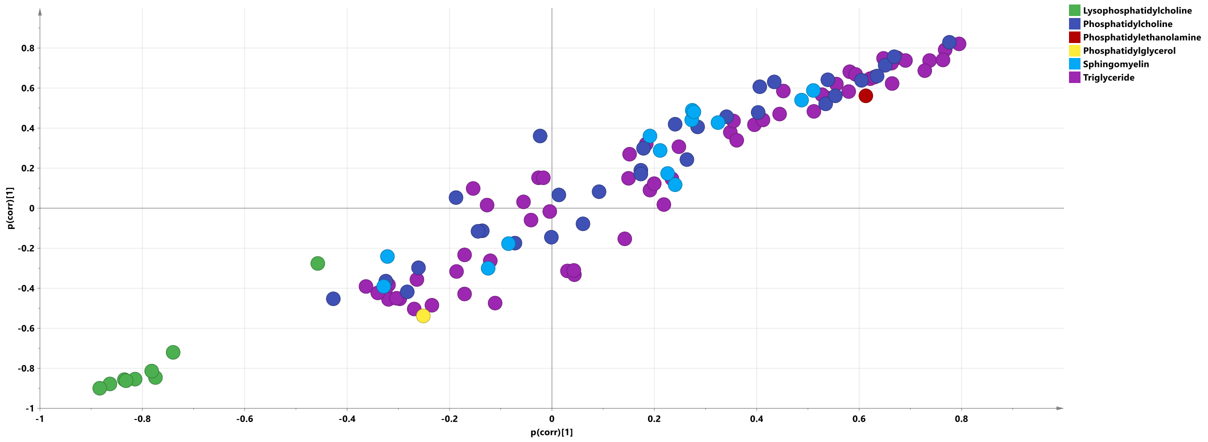

Figure S9. SUS-plot of OPLS-DA correlation loadings (p(corr)) from the mild versus controls (X axis) and severe versus controls (Y axis) models. With dots are symbolized lipid species marked in colors according to the chemical subclasses.

A.

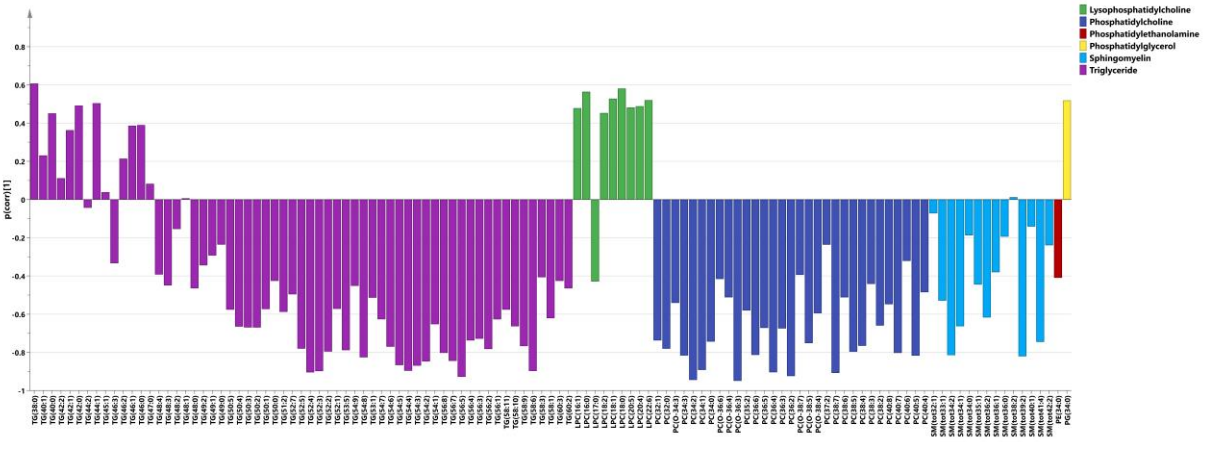
Lysophosphatidylcholine
Phosphatidylcholine
Phosphatidylethanolamine
Phosphatidylglycerol
Sphingomyelin
Triglyceride
p(corr)[1]

B.

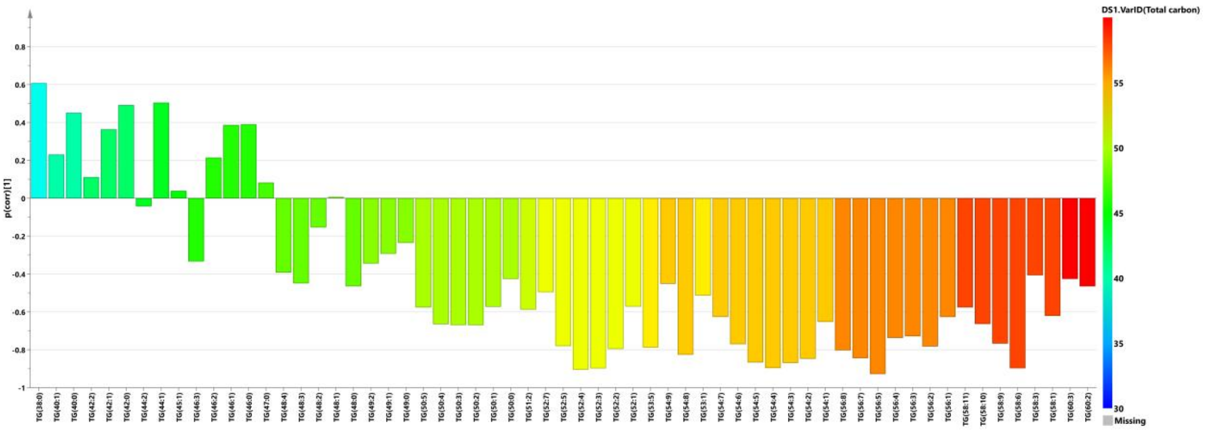
DS1.VarID(Total carbon)
p(corr)[1]
Missing

C.

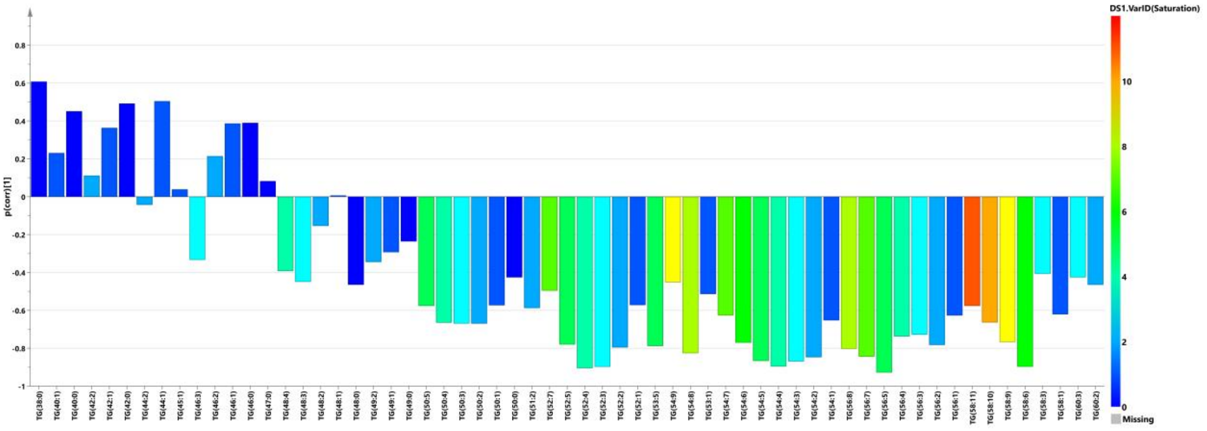

Fig. S10. Triglyceride and LPC subclass spectrum plots correlated to parasitaemia. A. Predictive loading vector (p(corr)) from the OPLS model for severe malaria samples with parasitaemia values as Y; lipid species are colored according to their chemical classes; p(corr) values indicate if the metabolite has a positive or negative correlation with parasitaemia values. B. Predictive loading vector (p(corr)) from the OPLS model for severe malaria samples with parasitaemia values as Y for triglycerides colored according to total carbon length of the triglyceride molecule. C. Predictive loading vector (p(corr)) from the OPLS model for severe malaria samples with parasitaemia values as Y for triglycerides colored according to total number of non-saturated locations in the triglyceride carbon chain.
